# Supplementary material for: Appropriate use of blood cultures in the emergency department through machine learning (ABC): study protocol for a randomised controlled non-inferiority trial
Source: BMJ Open. 2024 May 31;14(5):e084053. doi: 10.1136/bmjopen-2024-084053 (PMC11149153; doi:10.1136/bmjopen-2024-084053)
Supplement: Supplementary data [file bmjopen-2024-084053supp001.pdf]

Participant Information for Participation in Medical Scientific Research

The ABC Study – Appropriate Use of Blood Cultures in the Emergency Department through Machine Learning: a Randomized Controlled Trial.

Introduction

Dear Sir/Madam,

You are receiving this letter because you have visited the emergency department of Amsterdam UMC and your attending physician intends to collect a blood culture from you, or has already done so. With this information letter, we would like to ask you to participate in medical scientific research. Participation is voluntary. You will learn about the nature of the research, its implications for you, and the advantages and disadvantages involved. It is a lot of information. Would you please read through the information and decide if you wish to participate? If you decide to participate, you can fill out the form provided in the attachment.

Ask Your Questions

You can make your decision based on the information provided in this information letter. Additionally, we recommend the following:

- Ask questions to the researcher who provided you with this information letter.

- Discuss this research with your partner, family, or friends.

- Ask questions to an independent expert

- Read information on www.rijksoverheid.nl/mensenonderzoek.

1. General Information

Amsterdam UMC – location VUmc has initiated this research. Researchers, including physicians, medical students, researchers, and research nurses, conduct the research. The Central Committee on Research Involving Human Subjects (CCMO) has validated this research, and the Medical Ethics Review Committee (METc) has approved it as well. Participation in our scientific research is voluntary. However, we require your written consent to participate.

2. What is the Purpose of the Research?

The aim of this research is to reduce unnecessary diagnostics with blood cultures, since blood cultures can have various adverse effects. Therefore, in this study, we are investigating the safety and effectiveness of a computer program that predicts the outcome of a blood culture. This prediction model assists the physician in deciding whether a blood culture should be analyzed or not. If the probability of the blood culture yielding useful results is very low, we aim to cancel the blood culture analysis and prevent potential adverse effects for the patient.

van der Zaag AY, *et al. BMJ Open* 2024; 14:e084053. doi: 10.1136/bmjopen-2024-084053

3. What is the Background of the Research?

Blood cultures can detect bacteria in the bloodstream. Although bloodstream infections are not common, blood cultures are frequently used as part of standard care for patients with possible infections. In the emergency department, only about 10% of blood cultures yield positive results. The other 90% provide no information or even incorrect information (a bacterium is found, but it is not the cause of the infection). Patients with such "false-positive" cultures often receive unnecessary antibiotics, undergo more procedures, and on average stay in the hospital for a longer period of time.

Our research group has developed a computer program (prediction model) that can predict the likelihood of a positive blood culture. In this study, we aim to omit blood culture analysis when the chance of a positive result is very low, thereby avoiding unnecessary testing. By analyzing fewer blood cultures, we can also reduce the number of false-positive results. Consequently, unnecessary antibiotic administration, procedures, and hospital admissions can be avoided. In this study, we aim to determine whether this approach is indeed safe and assess the benefits for patients.

4. How Does the Research Proceed?

How long does the research last for?

If you participate in the research, your data will be reviewed for up to 3 months after your visit to the emergency department, using the electronic patient record.

Step 1: Are You Eligible to Participate?

We first need to determine if you are eligible to participate. Therefore, the researcher checks whether your attending physician indeed intends to collect a blood culture (or has already done so). The researcher also verifies if there are sufficient data available in your patient record for the prediction model to make a prediction.

In some cases, even if the above conditions apply to you, you may still not be eligible to participate in the research. This is the case, for example, if you recently have had a bloodstream infection.

Step 2: Using the Prediction Model

For this research, we have two groups, and the allocation to these groups is determined by randomization:

- Group 1 (intervention group): The physician of the individuals in this group uses the prediction model.
- Group 2 (control group): The physician of the individuals in this group does not use the prediction model.

Only in Group 1 can the prediction model be used to determine whether the blood culture should be analyzed or not.

How is it different from standard care?

If you are in the control group (Group 2) of the study, there is no difference from standard care. If you are assigned to Group 1 (the intervention group), your physician may cancel the analysis of your blood culture. Normally, the physician would proceed with the analysis. However, please note that your physician will only make this decision if the prediction model indicates a very low risk for a bloodstream infection. From that point onwards, the physician will consider the blood culture as "negative" and treat you accordingly.

5. What Agreements Do We Make with You?

We want the research to proceed smoothly. Therefore, we make the following agreement with you:

- You will contact the researcher if you no longer wish to participate in the research.

6. What Side Effects, Adverse Effects, or Inconveniences Can You Experience?

The use of the prediction model may lead the physician to not collect a blood culture from you, whereas it would have been done otherwise. Then, there is a very small chance that a bloodstream infection may be missed. Even if such an infection is missed, it is likely to be treated effectively, or you may recover spontaneously. However, in the worst-case scenario, there is a chance of severe harm or even death due to this infection, which could have been prevented by timely treatment based on the result of a blood culture.

7. What are the Benefits and Disadvantages of Participating in the Research?

If you are allocated to the intervention group, your blood culture analysis may be canceled. This reduces unnecessary testing for you. Not only does this save costs, but it may also result in receiving fewer or different antibiotics, undergoing fewer tests, and spending less time in the hospital.

Participating in the research may also have adverse consequences, as mentioned above in point 6. Research on our prediction model has shown that the risk of these adverse consequences is very small.

Do You Not Want to Participate?

You decide whether or not to participate in the research. If you choose not to participate, you will still receive the standard care you would otherwise receive.

8. When Does the Research End?

The researcher will inform you if there is new information about the research that is important for you. The researcher will then ask you if you wish to continue participating. In the following situations, the research ends for you:

- All examinations according to the schedule are completed.
- You decide to withdraw from the research yourself. You can do this at any time. Please inform the researcher immediately. You do not need to explain why you are withdrawing.
- One of the following authorities decides that the research must stop
    - Amsterdam UMC – location VUmc,
    - The government, or the medical ethics committee that assesses the research.

What Happens If You Withdraw from the Research?

Inform the researcher about this. The researchers will use the data collected up to the point of withdrawal but will not collect any new data.

The entire research is concluded when the data of all participants have been checked in the medical record after 3 months.

9. What Happens After the Research?

Approximately one year after the research is completed, the researchers will publish the results of the research in a medical scientific journal.

10. What Do We Do with Your Data?

If you participate in the research, you also consent to the collection, use, and storage of your data.

What data do we store?

We store this data:

- Your gender

- Your age

- Data about your health

At the end of the research, we will also request your data once from the Personal Records Database (BRP), so that we are informed if anything happens to you outside the hospital.

Why do we collect, use, and store your data?

We collect, use, and store your data to answer the questions of this research and to publish the results.

How do we protect your privacy?

To protect your privacy, we assign a code to your data. Only this code is used on all your data. We keep the key to the code in a secure location at Amsterdam UMC. When processing your data, we always use only this code. Even in reports and publications about the research, no one can tell that it was about you.

Who can see your data?

Some people may view your name and other personal data without a code. These are people who verify whether the researchers are conducting the research properly and reliably. These people can access your data:

• An auditor working for the researcher.

• National and international supervisory authorities. For example, the Healthcare and Youth Inspectorate.

These individuals keep your data confidential. We ask you to give consent for this access.

How long do we store your data?

We store your data for 15 years in the hospital, to analyze and publish the results.

Can you withdraw your consent for the use of your data?

You can withdraw your consent for the use of your data at any time. This applies to the use in this research and in other research. However, please note: if you withdraw your consent, and researchers have already collected data for a study, they may still use this data.

Can the researchers approach you again for follow-up research?

The researchers may approach you for follow-up research. You can indicate on the consent form whether you agree to this or not.

Do You Want to Know More About Your Privacy?

- Do you want to know more about your rights regarding the processing of personal data? Then visit www.autoriteitpersoonsgegevens.nl.
- Do you have questions about your rights? Or do you have a complaint about the processing of your personal data? Then contact the person responsible for processing your personal data. For your research, that is:
  - Amsterdam UMC
- If you have complaints about the processing of your personal data, we recommend that you first discuss this with the research team. You can also contact the Data Protection Officer of Amsterdam UMC. Or you can file a complaint with the Personal Data Authority.

11. Will You Receive Compensation for Participating in the Research?

You will not receive compensation for participating in this research.

12. Are You Insured During the Research?

Insurance has been taken out for everyone participating in this research. The insurance covers damage caused by the research. But not all damage. In Appendix B, you will find more information about the insurance and the exceptions. It also states to whom you can report damage.

13. Do You Have Questions?

You can ask questions about the research to the research team. If you want advice from someone with no vested interest, consult the independent expert. They are knowledgeable about the research but are not involved in conducting it.

Do you have a complaint? Discuss this with the researcher or the physician treating you. Prefer not to do this? Then contact the hospital's complaints officer. Appendix A provides information on where to find them.

14. How Do You Give Consent for the Research?

You can take time to consider this research. After an hour of consideration, you inform the researcher whether you understand the information and whether you wish to participate or not. If you choose to participate, you will fill out the consent form provided with this information letter. Both you and the researcher will receive a signed version of this consent statement.

Thank you for your time.

van der Zaag AY, *et al. BMJ Open* 2024; 14:e084053. doi: 10.1136/bmjopen-2024-084053

The ABC Study – 'Appropriate use of Blood Cultures in the emergency department through machine learning: a randomized controlled trial.'

I have read the participant information associated with the ABC study. I understand the information provided. My questions have been answered satisfactorily, and I had sufficient time to decide whether or not to participate in this study. I understand that my participation is entirely voluntary. I am aware that I can withdraw at any time without giving a reason.

• I consent to the use, storage, and processing of my encoded data for research purposes related to blood cultures.

• I consent to the retention of my encoded data for 15 years.

• I understand that for the monitoring of the research, some individuals may access all of my data. These individuals are listed in this information letter. I authorize these individuals to access my data for monitoring purposes.

Please indicate below yes or no:

I consent to potentially being asked to participate in a follow-up study after this research.

Yes ☐ No ☐

I consent to the retrieval of my data from the Personal Records Database (BRP).
Yes ☐ No ☐

Name: _______________________ Signature: _________________

Date: _______________________

I hereby declare that I have fully informed this participant about the ABC study. If any information arises during the study that may affect the participant's consent, I will inform them promptly.

Researcher's Name (or representative): _______________________

Signature: _________________

Date: _______________________

van der Zaag AY, *et al. BMJ Open* 2024; 14:e084053. doi: 10.1136/bmjopen-2024-084053
