# Supplementary material for: Appropriate use of blood cultures in the emergency department through machine learning (ABC): study protocol for a randomised controlled non-inferiority trial
Source: BMJ Open. 2024 May 31;14(5):e084053. doi: 10.1136/bmjopen-2024-084053 (PMC11149153; doi:10.1136/bmjopen-2024-084053)
Supplement: Author's manuscript [file bmjopen-2024-084053.draft_revisions.pdf]

BMJ Open is committed to open peer review. As part of this commitment we make the peer review history of every article we publish publicly available.

When an article is published we post the peer reviewers' comments and the authors' responses online. We also post the versions of the paper that were used during peer review. These are the versions that the peer review comments apply to.

The versions of the paper that follow are the versions that were submitted during the peer review process. They are not the versions of record or the final published versions. They should not be cited or distributed as the published version of this manuscript.

BMJ Open is an open access journal and the full, final, typeset and author-corrected version of record of the manuscript is available on our site with no access controls, subscription charges or pay-per-view fees (http://bmjopen.bmj.com).

If you have any questions on BMJ Open's open peer review process please email info.bmjopen@bmj.com

BMJ Open

Appropriate use of Blood Cultures in the emergency department through machine learning (ABC): study protocol for a randomized controlled non-inferiority trial

Journal:	*BMJ Open*
Manuscript ID	bmjopen-2024-084053
Article Type:	Protocol
Date Submitted by the Author:	07-Jan-2024

Complete List of Authors:	van der Zaag, Anuschka; Amsterdam UMC Locatie VUmc, Department of Internal Medicine, Division of Acute Medicine<br>Bhagirath, Sheena C.; Amsterdam UMC Locatie VUmc, Department of Internal Medicine, Division of Acute Medicine<br>Boerman, Anneroos; Amsterdam UMC Locatie VUmc, Department of Internal Medicine, Division of Acute Medicine; Amsterdam UMC Locatie VUmc, Department of Laboratory Medicine<br>Schinkel, Michiel; Amsterdam University Medical Centres, Center for Experimental and Molecular Medicine (C.E.M.M.); Amsterdam UMC Locatie VUmc, Department of Internal Medicine, Division of Acute Medicine<br>Paranjape, Ketan ; Amsterdam UMC Locatie VUmc, Department of Internal Medicine, Division of Acute Medicine<br>Azijli, Kaoutar; Amsterdam UMC Locatie VUmc, Department of Emergency Medicine<br>Lie, Mei; Amsterdam UMC Locatie VUmc, Department of EVA Service Center<br>Schade, Rogier; Amsterdam UMC Locatie VUmc, Department of Medical Microbiology and Infection Prevention<br>Nanayakkara, Prabath; Amsterdam UMC Locatie VUmc, Department of Internal Medicine, Division of Acute Medicine<br>Ridderikhof, Milan L. ; Amsterdam UMC Locatie AMC, Department of Emergency Medicine<br>Lissenberg-Witte, Birgit; Amsterdam UMC Locatie VUmc, Department of Epidemiology & Data Science<br>Wiersinga, Joost; Amsterdam UMC Locatie AMC, Department of Internal Medicine, Division of Infectious Diseases; Amsterdam UMC Locatie AMC, Center for Experimental and Molecular Medicine (C.E.M.M.)<br>de Jonge, Robert; Amsterdam UMC Locatie VUmc, Department of Laboratory Medicine

Keywords:	ACCIDENT & EMERGENCY MEDICINE, Clinical Decision-Making, INFECTIOUS DISEASES, Diagnostic microbiology < INFECTIOUS DISEASES, INTERNAL MEDICINE

SCHOLARONE™
Manuscripts

Appropriate use of Blood Cultures in the emergency department through machine learning (ABC): study protocol for a randomized controlled non-inferiority trial

Anuschka Y. van der Zaag[1], Sheena C. Bhagirath[1*], Anneroos W. Boerman[1,2], Michiel Schinkel[1,3], Ketan Paranjape[1], Kaoutar Azijli[4], Milan L. Ridderikhof[4], Mei Lie[5], Birgit Lissenberg-Witte[6], Rogier Schade[7], W. Joost Wiersinga[3,8], Robert de Jonge[2], Prabath W.B. Nanayakkara[1]*

[*] Both authors contributed equally to this paper.

[1]Department of Internal Medicine, Division of Acute Medicine, Amsterdam UMC, VU University, Amsterdam, The Netherlands.

[2]Department of Laboratory Medicine, Amsterdam UMC, Amsterdam, The Netherlands.

[3]Center for Experimental and Molecular Medicine (CEMM), Amsterdam UMC, University of Amsterdam, Amsterdam, The Netherlands.

[4]Department of Emergency Medicine, Amsterdam UMC, Amsterdam, The Netherlands

[5]Department of EVA Service Center, Amsterdam UMC, Amsterdam, The Netherlands

[6]Department of Epidemiology & Data Science, Amsterdam UMC, The Netherlands

[7]Department of Medical Microbiology and Infection Prevention, Amsterdam UMC, Amsterdam, the Netherlands

[8]Department of Internal Medicine, Division of Infectious Diseases, Amsterdam UMC, University of Amsterdam, Amsterdam, The Netherlands.

Corresponding author: Prabath WB Nanayakkara, Acute Medicine Section, Department of Internal Medicine, Amsterdam UMC location VUmc, De Boelelaan 1118, Amsterdam, 1081 HZ, Netherlands, Email: p.nanayakkara@amsterdamumc.nl

For peer review only

Abstract

Introduction

The liberal use of blood cultures in emergency departments leads to low yields and high numbers of false-positive results. False-positive, contaminated cultures are associated with prolonged hospital stays, increased antibiotic usage, and even higher hospital mortality rates. This trial aims to investigate whether a recently developed and validated machine learning model for predicting blood culture outcomes can safely and effectively guide clinicians in withholding unnecessary blood culture analysis.

Methods and analysis

A randomized controlled, non-inferiority trial comparing current practice with a machine learning guided approach. The primary objective is to determine whether the machine learning based approach is non-inferior to standard practice based on 30-day mortality. Secondary outcomes include hospital length-of stay, antibiotic usage, and model performance. Participants will be recruited in the emergency departments of multiple hospitals in the Netherlands. A total of 7584 participants will be included.

Ethics and dissemination

This study has been approved by the Amsterdam University Medical Centers' local medical ethics review committee (nr. 22.0567) and has been submitted for registration at Clinicaltrials.gov (NCT06163781). The study will be conducted in concordance with the principles of the Declaration of Helsinki, and in accordance with the Medical Research Involving Human Subjects Act, General Data Privacy Regulation, and Medical Device Regulation. Research results will be published in peer-reviewed journals.

Strengths and limitations of this study

- Pioneering the use of machine learning in a randomized controlled trial to predict blood culture outcomes.
- Conducting a comparative analysis against current clinical practices.
- Assessing patient-centered outcomes and performing an economic evaluation.
- May restrict future applications due to the exclusion of immunocompromised patients.

For peer review only

For peer review only

Introduction

Over 20% of adult emergency department visits occur due to infections [1]. Often, physicians will request blood cultures in these patients due to fear of a blood stream infection (BSI) [2]. Because of the liberal use of blood cultures, the yield tends to be low. Depending on the setting, only about 1-15% of blood cultures grow pathogens, of which 30-55% turn out to be false-positive, contaminated results [3-5]. Those contaminated cultures are associated with prolonged hospital stays, unnecessary use of antibiotics, and even hospital mortality [3, 6, 7]. To optimize blood culture use, it is crucial to better target which patients would benefit from blood culture analysis [8].

A machine learning model that can predict the outcome of blood cultures in the emergency department has been developed to address this clinical dilemma, aiming to reduce unnecessary tests in low-risk patients, and help avoid the harms associated with false-positive results due to blood culture contamination. The performance of the model has been internally and externally validated in various settings, including a real-time background evaluation in the Amsterdam University Medical Centers (Amsterdam UMC, locations VUmc and AMC) Electronic Health Records (EHR) system, where it maintained an Area Under the Operating Curve (AUC) of 0.76 and showed a potential reduction of approximately 30% blood cultures in the future [9, 10]. Additionally, the model has shown a consistent performance over time despite changes in clinical practice and patient characteristics [11].

The aim of this study is to investigate the safety and potential clinical benefits of using the blood culture prediction tool as decision support in reducing unnecessary blood cultures and thereby the negative side effects of false positive results for patients. This will be the first randomized controlled trial testing such a machine learning prediction tool in clinical practice.

For peer review only

Methods and analysis

This study protocol adheres to the Standard Protocol Items: Recommendations for Interventional Trials (SPIRIT) statement.

Study design

This study will be a multi-center randomized controlled non-inferiority trial. Participants will be randomized into an intervention and control arm. In the intervention group, the blood culture prediction model is used to predict the probability of a positive blood culture in the patient. The predictions made by the model will be shown to the physician after randomization in a dashboard integrated in the electronic health records (EHR). The blood culture analysis will be canceled if the probability of a positive culture is less than 5%. This will be done manually by the study team. If the probability is over or equal to 5%, the blood culture will be analyzed as per standard practice. In the control group, all patients will have their blood cultures analyzed. The study design is summarized in Figure 1. Patients will be randomized within the EHR system, to ensure that physicians cannot be influenced in their treatment decisions by seeing the score before the patient is included. Inclusions of patients will start in February 2024. Follow-up of the last patients is expected in February 2027.

Population

All adult patients presenting to the emergency departments (ED) of multiple hospitals in the Netherlands, and in whom blood cultures are ordered during ED stay will be assessed for eligibility. This process is automated within the EHR. The treating physician determines whether there is an indication for performing blood cultures according to the current local standards, after which both the patient and study team are informed about possible study recruitment. Inclusion and exclusion criteria are summarized in Box 1. Some patients will be excluded due to the higher possibility of BSIs with a pathogen generally considered a contaminant (e.g. a central line in situ) or because of the possibility of severe clinical implications of omitting blood cultures (e.g. severe neutropenia). Furthermore, patients unable to give informed consent and pregnant or breastfeeding patients will be excluded.

Sample size calculation

An analysis of 5907 unique ED visits with blood culture sampling in Amsterdam UMC showed a 30-day mortality rate of 7.6%, a hospital admission rate of 67.4%, and an average length of stay in the hospital of 6.7 days. Based on these numbers, the sample sizes with a relative non-inferiority margin of 1.25 for the rates and 1 day for the length of stay were calculated. The sample sizes were calculated by a statistician based on a one-sided alpha of 5% and a target power of at least 80%. For 30-day mortality, the calculated sample size was 6066 (3033 per arm) to test non-inferiority. After inflation for a dropout rate of 20%, the final sample size is 7584 (3792 per arm). For the secondary outcomes, described in Box 2, fewer participants were needed for adequate power.

For peer review only

Outcomes

The primary objective is to investigate whether using a machine learning based blood culture prediction tool is non-inferior to current practice regarding 30-day all-cause mortality. All secondary outcomes are described in Box 2. Model performance will be evaluated using the AUC, AUPRC and calibration for the model in the complete study population and in subgroups based on comorbidities. Additionally, patient reported outcomes will be included to determine if the model also influences patient-reported outcome measures such as side-effects from antibiotics and quality of life. For assessment of quality of life the EQ-5D-5L questionnaire [12] will be filled out at baseline and at 3 months. Lastly, a cost-effectiveness analysis will be performed using cost questionnaires based on the iMCQ and iPCQ [13]. A timeline visualizing data collection is shown in Figure 2.

Data collection and management

The data underlying this study, including the outcome measures such as mortality and admission rates, will be automatically extracted from the EHR system into an electronic data capture system (Castor EDC). The questionnaires regarding patient-reported outcomes and cost-benefit analysis will subsequently be sent out digitally through the EDC system. The data will be stored for 15 years, in agreement with local regulations and with consent.

Safety, adverse events, and monitoring

Adverse events will be monitored by the investigators, including device related adverse events. Serious adverse events will be reported within a maximum of 15 days to the local authority, and an annual safety report will be submitted to the local authorities. The study has its own data safety monitoring board (DSMB) consisting of clinicians, a statistician, and an epidemiologist, independent of the study team. During the study period, the DSMB will be supplied with an interim analysis, and will advise the study team based on these results. Additionally, the study will be monitored by an independent clinical trial monitoring committee, which performs quality checks on the data and regular on-site auditing.

Allocation and randomization

Participants will be randomly assigned to either the intervention or control group with a 1:1 allocation through a validated computer-generated random number within the EHR system. This will be a simple randomization without stratification. Randomization will occur automatically after consent and after blood cultures have been ordered. Physicians caring for a patient in the control group will not see any algorithm predictions. Physicians caring for a patient in the intervention group will be able to see the algorithm prediction. Due to the nature of the intervention, neither participant nor staff can be blinded to the allocation.

Statistical analysis

We will carry out a per-protocol analysis. The primary outcome, the 30-day mortality rate, will be compared between the groups using a non-inferiority test for the ratio of two proportions. Similar tests will be performed for in-hospital mortality rate and the hospital admission rate. The hospital length-of-stay will be analyzed as a

For peer review only

continuous variable and a non-inferiority test for the differences between the two means will be used. The test statistic to be used is a one-sided test with a significance level of 0.05. The model-related outcomes will be assessed using an area under the curve and calibration plots.

Patient and public partnership

Patient participation is important in both the clinical trial and the implementation of AI in clinical practice. The Client Advisory Board of Amsterdam UMC and a former sepsis patient evaluated the study question as relevant and the study design as achievable from patients' perspective. Also, to disseminate results of the study, we will collaborate with relevant patient organizations. Furthermore, a patient safety and implementation expert was involved in the development of the study protocol, and will remain involved during the study period. They will continue to stay involved if the study is deemed successful and the model can be implemented into clinical practice.

For peer review only

Ethics and dissemination

Obtaining informed consent is of the utmost importance when working with study participants. In order to ensure that participants are well informed about the study before consenting, all potential participants will have the trial clearly explained to them by a trained member of the study team. In addition to the verbal information, a paper information brochure will be provided to each participant. Each participant will be made aware of their right to withdraw consent at any given time. Due to the nature of the study, the potential participant will get 1 hour to consider joining the study.

This study will be conducted according to the principles of the Declaration of Helsinki, and in accordance with the Medical Research Involving Human Subjects Act, General Data Privacy Regulation, and Medical Device Regulation. The study was approved by the Amsterdam University Medical Center's medical ethics review committee with number 22.0567. The trial was registered at Clinicaltrials.gov (NCT06163781).

For peer review only

Conclusion

Blood cultures are a commonly used diagnostic tool in the ED. Unfortunately, low yields and high rates of contamination lead to unnecessary use of antibiotics, prolonged length-of-stay in the hospital, and even higher mortality. A machine learning model has been validated retrospectively and prospectively, and can identify patients at low risk for positive blood cultures. The study described in this protocol is the first randomized controlled trial using machine learning to predict blood culture outcomes. By using this model in practice, it can potentially reduce the amount of blood culture analyses and prevent the undesirable effects of false-positive blood culture results.

For peer review only

Statements

Authors' contributions

AZ and SB contributed equally to this paper. AZ and SB wrote the first draft of the paper. AB, MS and PN conceptualized the study. AB, MS, JW and PN secured funding for the study. BLW provided the sample size calculations and statistical analysis plan. ML developed the resources. KP, RS, KA, MR, ML, JW, RdJ and PN supervised various parts of the research process within their expertise. AZ, SB, AB, MS, MR and PN revised the manuscript. All authors approved the final version of the manuscript.

Funding statement

This RCT is funded by ZonMW DoelmatigheidsOnderzoek, grant number 2012379. Additionally, this trial is supported by a TKI grant, which aims to use these trial results to further optimize the tool. The funders had no role in the study design, and will have no role in its execution, data analyses or decision to publish.

Competing interests

All authors have nothing to disclose.

For peer review only

References

1. Wang, H.E., A.R. Jones, and J.P. Donnelly, *Revised National Estimates of Emergency Department Visits for Sepsis in the United States.* Crit Care Med, 2017. **45**(9): p. 1443-1449.
2. Fabre, V., et al., *Prescribers' knowledge, attitudes and perceptions about blood culturing practices for adult hospitalized patients: a call for action.* Infect Control Hosp Epidemiol, 2018. **39**(11): p. 1394-1396.
3. Long, B. and A. Koyfman, *Best Clinical Practice: Blood Culture Utility in the Emergency Department.* J Emerg Med, 2016. **51**(5): p. 529-539.
4. Nannan Panday, R.S., et al., *Evaluation of blood culture epidemiology and efficiency in a large European teaching hospital.* PLoS One, 2019. **14**(3): p. e0214052.
5. Denny, K.J., et al., *Is it time for a culture change? Blood culture collection in the emergency department.* Emerg Med Australas, 2018. **30**(4): p. 575-577.
6. Klucher, J.M., et al., *Risk factors and clinical outcomes associated with blood culture contamination.* Infect Control Hosp Epidemiol, 2022. **43**(3): p. 291-297.
7. Doern, G.V., et al., *Practical Guidance for Clinical Microbiology Laboratories: A Comprehensive Update on the Problem of Blood Culture Contamination and a Discussion of Methods for Addressing the Problem.* Clin Microbiol Rev, 2019. **33**(1).
8. Fabre, V., et al., *Does This Patient Need Blood Cultures? A Scoping Review of Indications for Blood Cultures in Adult Nonneutropenic Inpatients.* Clin Infect Dis, 2020. **71**(5): p. 1339-1347.
9. Boerman, A.W., et al., *Using machine learning to predict blood culture outcomes in the emergency department: a single-centre, retrospective, observational study.* BMJ Open, 2022. **12**(1): p. e053332.
10. Schinkel, M., et al., *Diagnostic stewardship for blood cultures in the emergency department: A multicenter validation and prospective evaluation of a machine learning prediction tool.* EBioMedicine, 2022. **82**: p. 104176.
11. Schinkel, M., et al., *Detecting changes in the performance of a clinical machine learning tool over time.* EBioMedicine, 2023. **97**: p. 104823.
12. Herdman, M., et al., *Development and preliminary testing of the new five-level version of EQ-5D (EQ-5D-5L).* Qual Life Res, 2011. **20**(10): p. 1727-36.
13. Bouwmans, C., et al., *The iMTA Productivity Cost Questionnaire: A Standardized Instrument for Measuring and Valuing Health-Related Productivity Losses.* Value Health, 2015. **18**(6): p. 753-8.

For peer review only

CONSORT flow diagram

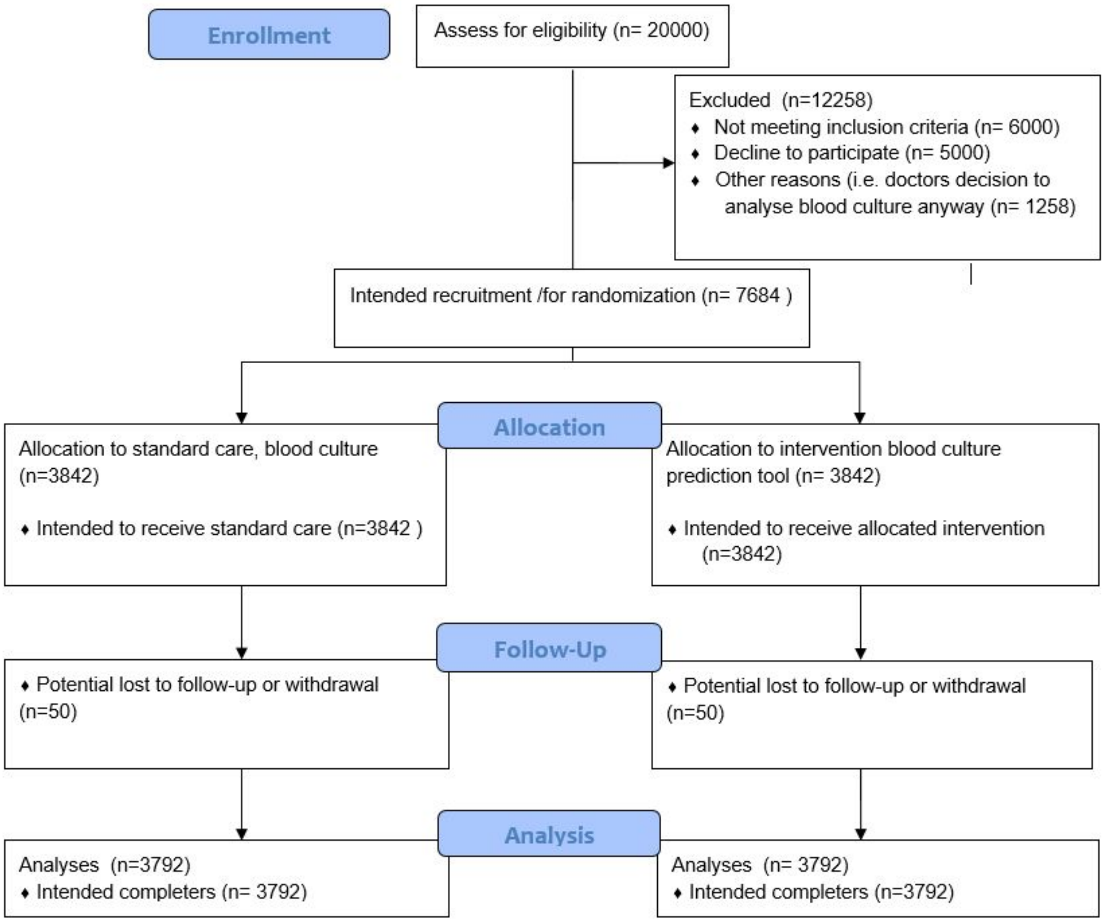

only

Figure 1

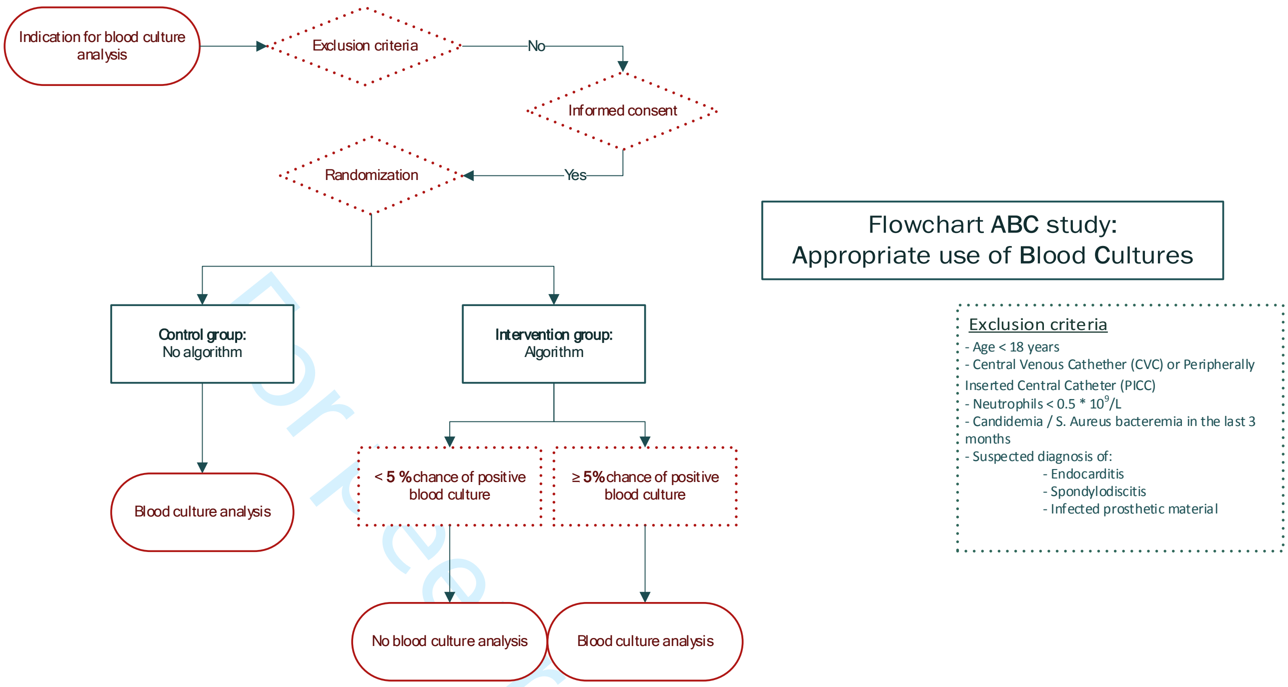

Figure 2

Allocation	Enrol ment	Allocation	Baseline	During admission*	3 months after baseline
Eligibility screen	X				
Informed consent	X				
Allocation		X			
Vital parameters			X		
Lab values			X		
Quality of life (according to EQ-5D-5L)			X		X
Canceling blood culture[A]			X[A]		
Admission rates			X		
Mortality				X	X
Patient-related outcomes[B]			X	X	X
Diagnostics related outcomes			X	X	X
Therapy-related outcomes			X	X	X
Model related outcomes			X	X	X
Cost questionnaire					X

*not applicable to all participants

A only in intervention group when predicted probability of positive blood culture is < 5%.

B see box 2

Box 1

Inclusion criteria:

- Age ≥ 18 years
- Willing and able to give informed consent to participate
- Have a clinical indication for blood culture analysis (according to the treating physician)
- Have sufficient data recorded (laboratory results and vital sign measurements) for a prediction to be made*

* Predictions can only be made when at least 20% of the needed parameters are available for a patient, which is the case in approximately 80% of all patients who undergo blood culture analyses in the ED.

Exclusion criteria:

- Patients with a Central Venous Catheter (CVC) or Peripherally Inserted Central Catheter (PICC) in situ
- Neutrophil count < 0.5 * 10^9/L
- Candidemia or *S. aureus* bacteremia in the past 3 months
- Suspected endocarditis, spondylodiscitis or infected prosthetic material
- Pregnant or breastfeeding patients
- Inability to give informed consent

Box 2

Primary outcome:

- 30-day all-cause mortality

Patient-related outcomes:

- In-hospital mortality
- 90-day mortality
- 30-day readmission rates
- Hospital admission rates
- Hospital length-of-stay in days
- Emergency department length-of-stay in hours

Diagnostics related outcomes:

- Percentage of blood cultures avoided in the intervention group
- Number of blood cultures on each day of hospital stay (in admitted patients)
- Percentage of positive blood cultures in each group
- Total number of laboratory- and microbiology tests in the ED
- Total number of laboratory- and microbiology test on each day of hospital stay (in admitted patients)

Therapy-related:

- Percentage of patients receiving antibiotics in the ED

For peer review only

- Duration of antibiotic therapy
- Types of antibiotics given in the ED

Model related:

- Model performance (AUC) during the trial
- Model performance in subgroups
    - Immunocompromised patients (triple immunosuppressive therapy)
    - Transplanted patients

For peer review only

Bijlage: CONSORT Flow-diagram

CONSORT Flow Diagram ABC Study

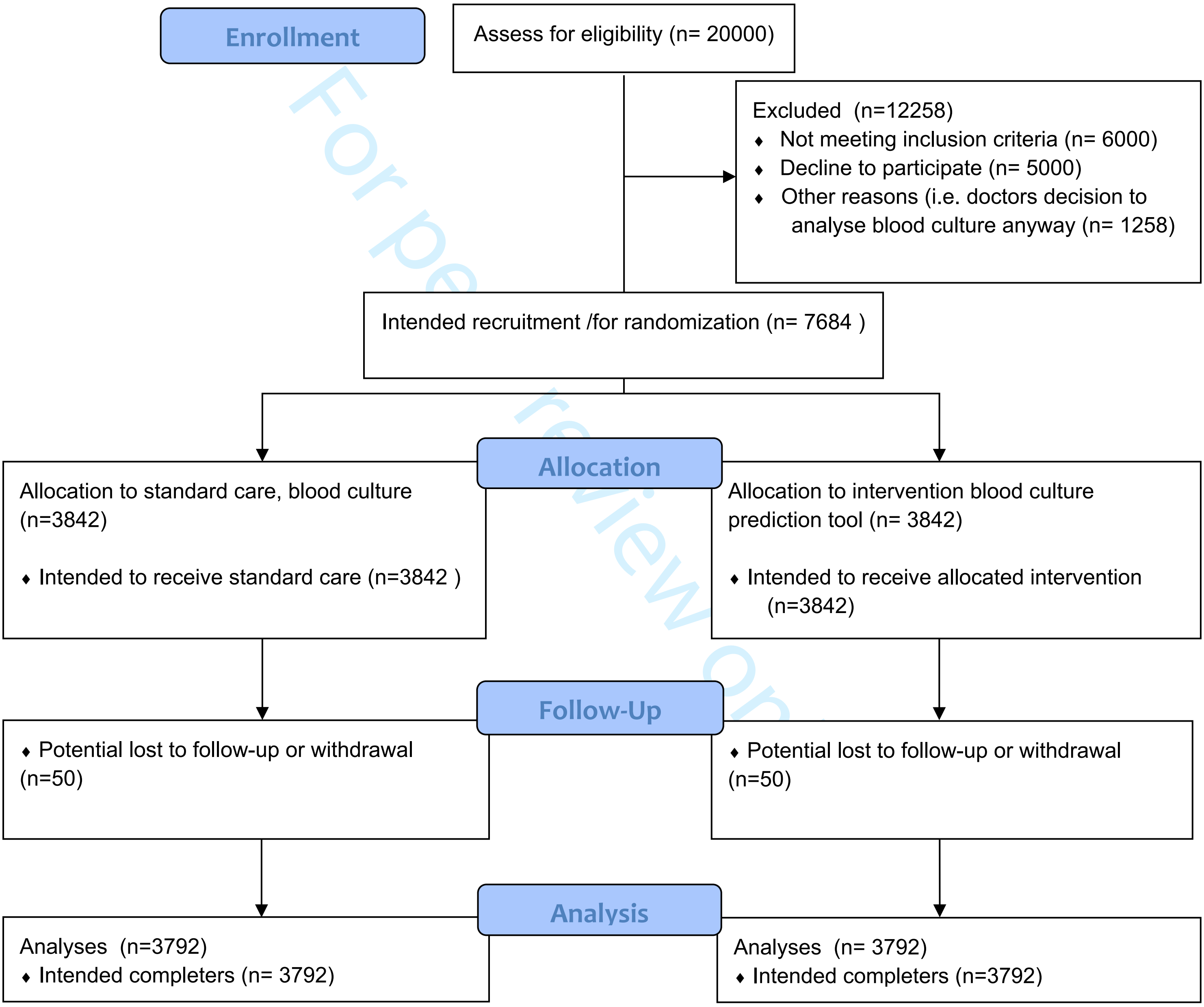

For peer review only - http://bmjopen.bmj.com/site/about/guidelines.xhtml

STANDARD PROTOCOL ITEMS: RECOMMENDATIONS FOR INTERVENTIONAL TRIALS

SPIRIT 2013 Checklist: Recommended items to address in a clinical trial protocol and related documents*

Section/item	Item No	Description
**Administrative information**		
Title	1	Descriptive title identifying the study design, population, interventions, and, if applicable, trial acronym
Trial registration	2a	Trial identifier and registry name. If not yet registered, name of intended registry
	2b	All items from the World Health Organization Trial Registration Data Set
Protocol version	3	Date and version identifier
Funding	4	Sources and types of financial, material, and other support
Roles and responsibilities	5a	Names, affiliations, and roles of protocol contributors
	5b	Name and contact information for the trial sponsor
	5c	Role of study sponsor and funders, if any, in study design; collection, management, analysis, and interpretation of data; writing of the report; and the decision to submit the report for publication, including whether they will have ultimate authority over any of these activities
	5d	Composition, roles, and responsibilities of the coordinating centre, steering committee, endpoint adjudication committee, data management team, and other individuals or groups overseeing the trial, if applicable (see Item 21a for data monitoring committee)
**Introduction**		
Background and rationale	6a	Description of research question and justification for undertaking the trial, including summary of relevant studies (published and unpublished) examining benefits and harms for each intervention
	6b	Explanation for choice of comparators
Objectives	7	Specific objectives or hypotheses
Trial design	8	Description of trial design including type of trial (eg, parallel group, crossover, factorial, single group), allocation ratio, and framework (eg, superiority, equivalence, noninferiority, exploratory)

For peer review only

Methods: Participants, interventions, and outcomes

Study setting	9	Description of study settings (eg, community clinic, academic hospital) and list of countries where data will be collected. Reference to where list of study sites can be obtained
Eligibility criteria	10	Inclusion and exclusion criteria for participants. If applicable, eligibility criteria for study centres and individuals who will perform the interventions (eg, surgeons, psychotherapists)
Interventions	11a	Interventions for each group with sufficient detail to allow replication, including how and when they will be administered
	11b	Criteria for discontinuing or modifying allocated interventions for a given trial participant (eg, drug dose change in response to harms, participant request, or improving/worsening disease)
	11c	Strategies to improve adherence to intervention protocols, and any procedures for monitoring adherence (eg, drug tablet return, laboratory tests)
	11d	Relevant concomitant care and interventions that are permitted or prohibited during the trial
Outcomes	12	Primary, secondary, and other outcomes, including the specific measurement variable (eg, systolic blood pressure), analysis metric (eg, change from baseline, final value, time to event), method of aggregation (eg, median, proportion), and time point for each outcome. Explanation of the clinical relevance of chosen efficacy and harm outcomes is strongly recommended
Participant timeline	13	Time schedule of enrolment, interventions (including any run-ins and washouts), assessments, and visits for participants. A schematic diagram is highly recommended (see Figure)
Sample size	14	Estimated number of participants needed to achieve study objectives and how it was determined, including clinical and statistical assumptions supporting any sample size calculations
Recruitment	15	Strategies for achieving adequate participant enrolment to reach target sample size

Methods: Assignment of interventions (for controlled trials)

Allocation:

Sequence generation	16a	Method of generating the allocation sequence (eg, computer-generated random numbers), and list of any factors for stratification. To reduce predictability of a random sequence, details of any planned restriction (eg, blocking) should be provided in a separate document that is unavailable to those who enrol participants or assign interventions

Allocation concealment mechanism	16b	Mechanism of implementing the allocation sequence (eg, central telephone; sequentially numbered, opaque, sealed envelopes), describing any steps to conceal the sequence until interventions are assigned
Implementation	16c	Who will generate the allocation sequence, who will enrol participants, and who will assign participants to interventions
Blinding (masking)	17a	Who will be blinded after assignment to interventions (eg, trial participants, care providers, outcome assessors, data analysts), and how
	17b	If blinded, circumstances under which unblinding is permissible, and procedure for revealing a participant's allocated intervention during the trial
**Methods: Data collection, management, and analysis**		
Data collection methods	18a	Plans for assessment and collection of outcome, baseline, and other trial data, including any related processes to promote data quality (eg, duplicate measurements, training of assessors) and a description of study instruments (eg, questionnaires, laboratory tests) along with their reliability and validity, if known. Reference to where data collection forms can be found, if not in the protocol
	18b	Plans to promote participant retention and complete follow-up, including list of any outcome data to be collected for participants who discontinue or deviate from intervention protocols
Data management	19	Plans for data entry, coding, security, and storage, including any related processes to promote data quality (eg, double data entry; range checks for data values). Reference to where details of data management procedures can be found, if not in the protocol
Statistical methods	20a	Statistical methods for analysing primary and secondary outcomes. Reference to where other details of the statistical analysis plan can be found, if not in the protocol
	20b	Methods for any additional analyses (eg, subgroup and adjusted analyses)
	20c	Definition of analysis population relating to protocol non-adherence (eg, as randomised analysis), and any statistical methods to handle missing data (eg, multiple imputation)
**Methods: Monitoring**		
Data monitoring	21a	Composition of data monitoring committee (DMC); summary of its role and reporting structure; statement of whether it is independent from the sponsor and competing interests; and reference to where further details about its charter can be found, if not in the protocol. Alternatively, an explanation of why a DMC is not needed

	21b	Description of any interim analyses and stopping guidelines, including who will have access to these interim results and make the final decision to terminate the trial
Harms	22	Plans for collecting, assessing, reporting, and managing solicited and spontaneously reported adverse events and other unintended effects of trial interventions or trial conduct
Auditing	23	Frequency and procedures for auditing trial conduct, if any, and whether the process will be independent from investigators and the sponsor
**Ethics and dissemination**		
Research ethics approval	24	Plans for seeking research ethics committee/institutional review board (REC/IRB) approval
Protocol amendments	25	Plans for communicating important protocol modifications (eg, changes to eligibility criteria, outcomes, analyses) to relevant parties (eg, investigators, REC/IRBs, trial participants, trial registries, journals, regulators)
Consent or assent	26a	Who will obtain informed consent or assent from potential trial participants or authorised surrogates, and how (see Item 32)
	26b	Additional consent provisions for collection and use of participant data and biological specimens in ancillary studies, if applicable
Confidentiality	27	How personal information about potential and enrolled participants will be collected, shared, and maintained in order to protect confidentiality before, during, and after the trial
Declaration of interests	28	Financial and other competing interests for principal investigators for the overall trial and each study site
Access to data	29	Statement of who will have access to the final trial dataset, and disclosure of contractual agreements that limit such access for investigators
Ancillary and post-trial care	30	Provisions, if any, for ancillary and post-trial care, and for compensation to those who suffer harm from trial participation
Dissemination policy	31a	Plans for investigators and sponsor to communicate trial results to participants, healthcare professionals, the public, and other relevant groups (eg, via publication, reporting in results databases, or other data sharing arrangements), including any publication restrictions
	31b	Authorship eligibility guidelines and any intended use of professional writers
	31c	Plans, if any, for granting public access to the full protocol, participant-level dataset, and statistical code

For peer review only

For peer review only - http://bmjopen.bmj.com/site/about/guidelines.xhtml

Appendices

Informed consent materials	32	Model consent form and other related documentation given to participants and authorised surrogates
Biological specimens	33	Plans for collection, laboratory evaluation, and storage of biological specimens for genetic or molecular analysis in the current trial and for future use in ancillary studies, if applicable

*It is strongly recommended that this checklist be read in conjunction with the SPIRIT 2013 Explanation & Elaboration for important clarification on the items. Amendments to the protocol should be tracked and dated. The SPIRIT checklist is copyrighted by the SPIRIT Group under the Creative Commons "Attribution-NonCommercial-NoDerivs 3.0 Unported" license.

For peer review only

BMJ Open

Appropriate use of Blood Cultures in the emergency department through machine learning (ABC): study protocol for a randomized controlled non-inferiority trial

Journal:	*BMJ Open*
Manuscript ID	bmjopen-2024-084053.R1
Article Type:	Protocol
Date Submitted by the Author:	20-Mar-2024
Complete List of Authors:	van der Zaag, Anuschka; Amsterdam UMC Locatie VUmc, Department of Internal Medicine, Division of Acute Medicine<br>Bhagirath, Sheena C.; Amsterdam UMC Locatie VUmc, Department of Internal Medicine, Division of Acute Medicine<br>Boerman, Anneroos; Amsterdam UMC Locatie VUmc, Department of Internal Medicine, Division of Acute Medicine; Amsterdam UMC Locatie VUmc, Department of Laboratory Medicine<br>Schinkel, Michiel; Amsterdam University Medical Centres, Center for Experimental and Molecular Medicine (C.E.M.M.); Amsterdam UMC Locatie VUmc, Department of Internal Medicine, Division of Acute Medicine<br>Paranjape, Ketan ; Amsterdam UMC Locatie VUmc, Department of Internal Medicine, Division of Acute Medicine<br>Azijli, Kaoutar; Amsterdam UMC Locatie VUmc, Department of Emergency Medicine<br>Ridderikhof, Milan L. ; Amsterdam UMC Locatie AMC, Department of Emergency Medicine<br>Lie, Mei; Amsterdam UMC Locatie VUmc, Department of EVA Service Center<br>Lissenberg-Witte, Birgit; Amsterdam UMC Locatie VUmc, Department of Epidemiology & Data Science<br>Schade, Rogier; Amsterdam UMC Locatie VUmc, Department of Medical Microbiology and Infection Prevention<br>Wiersinga, Joost; Amsterdam UMC Locatie AMC, Department of Internal Medicine, Division of Infectious Diseases; Amsterdam UMC Locatie AMC, Center for Experimental and Molecular Medicine (C.E.M.M.)<br>de Jonge, Robert; Amsterdam UMC Locatie VUmc, Department of Laboratory Medicine<br>Nanayakkara, Prabath; Amsterdam UMC Locatie VUmc, Department of Internal Medicine, Section General Internal Medicine
**Primary Subject Heading**:	Emergency medicine
Secondary Subject Heading:	Infectious diseases, Health informatics
Keywords:	ACCIDENT & EMERGENCY MEDICINE, Clinical Decision-Making, INFECTIOUS DISEASES, Diagnostic microbiology < INFECTIOUS DISEASES, INTERNAL MEDICINE

SCHOLARONE™
Manuscripts

Appropriate use of Blood Cultures in the emergency department through machine learning (ABC): study protocol for a randomized controlled non-inferiority trial

Anuschka Y. van der Zaag[1], Sheena C. Bhagirath[1*], Anneroos W. Boerman[1,2], Michiel Schinkel[1,3], Ketan Paranjape[1], Kaoutar Azijli[4], Milan L. Ridderikhof[4], Mei Lie[5], Birgit Lissenberg-Witte[6], Rogier Schade[7], W. Joost Wiersinga[3,8], Robert de Jonge[2], Prabath W.B. Nanayakkara[1]*

[*] Both authors contributed equally to this paper.

[1]Department of Internal Medicine, Section General Internal Medicine, Amsterdam UMC, VU University, Amsterdam, The Netherlands.

[2]Department of Laboratory Medicine, Amsterdam UMC, Amsterdam, The Netherlands.

[3]Center for Experimental Molecular Medicine (CEMM), Amsterdam UMC, University of Amsterdam, Amsterdam, The Netherlands.

[4]Department of Emergency Medicine, Amsterdam UMC, Amsterdam, The Netherlands

[5] Department of EVA Service Center, Amsterdam UMC, Amsterdam, The Netherlands

[6] Department of Epidemiology & Data Science, Amsterdam UMC, The Netherlands

[7] Department of Medical Microbiology and Infection Prevention, Amsterdam UMC, Amsterdam, the Netherlands

[8] Department of Internal Medicine, Division of Infectious Diseases, Amsterdam UMC, University of Amsterdam, Amsterdam, The Netherlands.

Corresponding author: Prabath WB Nanayakkara, Section General Internal Medicine, Department of Internal Medicine, Amsterdam UMC location VUmc, De Boelelaan 1118, Amsterdam, 1081 HZ, Netherlands, Email: p.nanayakkara@amsterdamumc.nl

For peer review only - http://bmjopen.bmj.com/site/about/guidelines.xhtml

Abstract

Introduction

The liberal use of blood cultures in emergency departments leads to low yields and high numbers of false-positive results. False-positive, contaminated cultures are associated with prolonged hospital stays, increased antibiotic usage, and even higher hospital mortality rates. This trial aims to investigate whether a recently developed and validated machine learning model for predicting blood culture outcomes can safely and effectively guide clinicians in withholding unnecessary blood culture analysis.

Methods and analysis

A randomized controlled, non-inferiority trial comparing current practice with a machine learning guided approach. The primary objective is to determine whether the machine learning based approach is non-inferior to standard practice based on 30-day mortality. Secondary outcomes include hospital length-of stay, antibiotic usage, and model performance. Participants will be recruited in the emergency departments of multiple hospitals in the Netherlands. A total of 7584 participants will be included.

Ethics and dissemination

Possible participants will receive verbal information and a paper information brochure regarding the trial. They will be given at least one hour consideration time before providing informed consent. Research results will be published in peer-reviewed journals. This study has been approved by the Amsterdam University Medical Centers' local medical ethics review committee (nr. 22.0567). The study will be conducted in concordance with the principles of the Declaration of Helsinki, and in accordance with the Medical Research Involving Human Subjects Act, General Data Privacy Regulation, and Medical Device Regulation.

Trial registration: ClinicalTrials.gov, NCT06163781.

For peer review only

Strengths and limitations of this study

- Pioneering the use of machine learning in a randomized controlled trial to predict blood culture outcomes.
- Conducting a comparative analysis against current clinical practices.
- Assessing patient-centered outcomes and performing an economic evaluation.
- May restrict future applications due to the exclusion of immunocompromised patients.

For peer review only - http://bmjopen.bmj.com/site/about/guidelines.xhtml

For peer review only

For peer review only - http://bmjopen.bmj.com/site/about/guidelines.xhtml

Introduction

Over 20% of adult emergency department visits occur due to infections [1]. Often, physicians will request blood cultures in these patients due to fear of a blood stream infection (BSI) [2]. Because of the liberal use of blood cultures, the yield tends to be low. Depending on the setting, only about 1-15% of blood cultures grow pathogens, of which 30-55% turn out to be false-positive, contaminated results [3-5]. Those contaminated cultures are associated with prolonged hospital stays, unnecessary use of antibiotics, and even hospital mortality [3, 6, 7]. To optimize blood culture use, it is crucial to better target which patients would benefit from blood culture analysis [8].

A machine learning model based on extreme gradient-boosting (XG Boost) that can predict the outcome of blood cultures in the emergency department has been developed to address this clinical dilemma, aiming to reduce unnecessary tests in low-risk patients, and help avoid the harms associated with false-positive results due to blood culture contamination [9, 10]. The performance of the model has been internally and externally validated in various settings, including a real-time background evaluation in the Amsterdam University Medical Centers (Amsterdam UMC, locations VUmc and AMC) Electronic Health Records (EHR) system, where it maintained an Area Under the Operating Curve (AUC) of 0.76 and showed a potential reduction of approximately 30% blood cultures in the future [9, 10]. Additionally, the model has shown a consistent performance over time despite changes in clinical practice and patient characteristics [11].

The aim of this study is to investigate the safety and potential clinical benefits of using the blood culture prediction tool as decision support in reducing unnecessary blood cultures and thereby the negative side effects of false positive results for patients. This will be the first randomized controlled trial testing such a machine learning prediction tool in clinical practice.

For peer review only

Methods and analysis

This study protocol adheres to the Standard Protocol Items: Recommendations for Interventional Trials (SPIRIT) statement.

Model development and validation

This segment features a summary of the model development, more detailed descriptions have been published previously [9, 10]. For the development of the model data from the VUmc was split into a training (80%) and a test set (20%), stratified by blood culture outcomes. Missing data was imputed by medians. The model was trained on the training set and subsequently validated in the VUmc test set, and three other hospital datasets (AMC, ZMC and BIDMC). These hospitals differ in size and population, comprising two tertiary teaching hospitals and one secondary care hospital in the Netherlands, along with one hospital dataset from the United States. Part of the data was gathered during the COVID-19 pandemic, but this did not affect its performance. The model contains 49 variables, namely age, sex, six vital sign measurements, and eighteen laboratory tests. Additionally, indicator variables of the laboratory tests were included. Figure 1 shows the 20 most important variables. The Area Under the Receiver Operating Characteristic (AUROC) Curves in the various validation cohorts ranged between 0.75-0.81. A prospective validation was also performed, integrating the model into the EHR of VUmc, showing an AUROC of 0.76. With the threshold for blood culture analysis set at 5%, analyses could be avoided in approximately 30%.

Study design

This study will be a multi-center randomized controlled non-inferiority trial. Participants will be randomized into an intervention and control arm. In the intervention group, the blood culture prediction model is used to predict the probability of a positive blood culture in the patient. The model will start predicting when sufficient variables are available in the EHR. A new prediction will be made every 20 minutes until 3 hours have passed since check—in of the patient in the ED. The predictions made by the model will be shown to the physician after randomization in a dashboard integrated in the electronic health records (EHR). The blood culture analysis will be canceled if the probability of a positive culture is less than 5%. This will be done manually by the study team. If the probability is over or equal to 5%, the blood culture will be analyzed as per standard practice. In the control group, all patients will have their blood cultures analyzed. The study design is summarized in Figure 2 and Figure 3. Patients will be randomized within the EHR system, to ensure that physicians cannot be influenced in their treatment decisions by seeing the score before the patient is included. The first patient was included in February 2024. Follow-up of the last patients is expected in February 2027.

Population

All adult patients (aged 18 years or older) presenting to the emergency departments (ED) of multiple hospitals in the Netherlands, and in whom blood cultures are ordered during ED stay will be assessed for eligibility. This process is automated within the EHR. The treating physician determines whether there is an indication for performing blood cultures according to the current local standards, after which both the patient and study team

For peer review only

are informed about possible study recruitment. Inclusion and exclusion criteria are summarized in Box 1. Some patients will be excluded due to the higher possibility of BSIs with a pathogen generally considered a contaminant (e.g. a central line in situ) or because of the possibility of severe clinical implications of omitting blood cultures (e.g. severe neutropenia). Since the study's goal is to reduce unnecessary testing, patients for whom blood culture analysis is deemed imperative are excluded (e.g. patients with a suspected diagnosis of endocarditis, spondylodiscitis or infected prosthetic material). Furthermore, patients unable to give informed consent and pregnant or breastfeeding patients will be excluded, due to them being considered high-risk groups by the ethics review board. Therefore, the review board did not give permission to include these groups.

**Box 1: Overview of inclusion and exclusion criteria**	
**Inclusion criteria**	Age ≥ 18 years
	Willing and able to give informed consent to participate
	Have a clinical indication for blood culture analysis (according to the treating physician)
	Have sufficient data recorded (laboratory results and vital sign measurements) for a prediction to be made*
**Exclusion criteria**	Patients with a Central Venous Catheter (CVC) or Peripherally Inserted Central Catheter (PICC) in situ
	Neutrophil count < 0.5 * 109/L
	Candidemia or S. aureus bacteremia in the past 3 months
	Suspected endocarditis, spondylodiscitis or infected prosthetic material
	Pregnant or breastfeeding patients
	Inability to give informed consent

Sample size calculation

An analysis of 5907 unique ED visits with blood culture sampling in Amsterdam UMC showed a 30-day mortality rate of 7.6%, a hospital admission rate of 67.4%, and an average length of stay in the hospital of 6.7 days. Based on these numbers, the sample sizes with a relative non-inferiority margin of 1.25 for the rates and 1 day for the length of stay were calculated. The sample sizes were calculated by a statistician based on a one-sided alpha of 5% and a target power of at least 80%. For 30-day mortality, the calculated sample size was 6066 (3033 per arm) to test non-inferiority. After inflation for a dropout rate of 20%, the final sample size is 7584 (3792 per arm). For the secondary outcomes, described in Box 2, fewer participants were needed for adequate power.

For peer review only - http://bmjopen.bmj.com/site/about/guidelines.xhtml

Recruitment

In order to achieve adequate participant enrolment, an announcement regarding the study is built into the EHR system. When the physician initiates a blood culture request in the EHR for an adult patient in the ED, a pop-up notification will automatically appear. This pop-up briefly outlines the study and presents both inclusion and exclusion criteria. If the patient provides permission to receive information about the study, the study team will be alerted and will provide the patient with additional information and obtain informed consent.

Outcomes

The primary objective is to investigate whether using a machine learning based blood culture prediction tool is non-inferior to current practice regarding 30-day all-cause mortality. All secondary outcomes are described in Box 2. Model performance will be evaluated using the AUROC, AUPRC and calibration for the model in the complete study population and in subgroups based on comorbidities. This will be assessed every three months so model performance over time can be guaranteed. The model will also continue to undergo prospective validation in the background of the EHR. Additionally, patient reported outcomes will be included to determine if the model also influences patient-reported outcome measures such as side-effects from antibiotics and quality of life. For assessment of quality of life the EQ-5D-5L questionnaire [12] will be filled out at baseline and at 3 months. Lastly, a cost-effectiveness analysis will be performed using cost questionnaires based on the iMCQ and iPCQ [13]. A timeline visualizing data collection is shown in Table 1.

**Box 2: Overview of outcome measures**	
**Primary outcome**	30-day all cause mortality
**Key secondary outcomes**	In-hospital mortality
	Hospital admission rates
	Hospital length-of-stay
**Other outcome measures**	
**Patient-related outcomes**	90-day mortality
	30-day readmission rates
	Emergency department length-of-stay in hours
**Diagnostics related outcomes**	Percentage of blood cultures avoided in the intervention group
	Number of blood cultures on each day of hospital stay (in admitted patients)
	Percentage of positive blood cultures in each group
	Total number of laboratory- and microbiology tests in the ED
	Total number of laboratory- and microbiology test on each day of hospital stay (in admitted patients)
**Therapy-related outcomes**	Percentage of patients receiving antibiotics in the ED
	Duration of antibiotic therapy
	Types of antibiotics given in the ED
**Model-related outcomes**	Model performance (AUC) during the trial
	Model performance in subgroups:<br>• Immunocompromised patients (triple immunosuppressive therapy)<br>• Transplanted patients

For peer review only - http://bmjopen.bmj.com/site/about/guidelines.xhtml

Table 1

Allocation	Enrol ment	Allocation	Baseline	During admission*	3 months after baseline
Eligibility screen	X				
Informed consent	X				
Allocation		X			
Vital parameters			X		
Lab values			X		
Quality of life (according to EQ-5D-5L)			X		X
Canceling blood culture[A]			X[A]		
Admission rates			X		
Mortality				X	X
Patient-related outcomes[B]			X	X	X
Diagnostics related outcomes			X	X	X
Therapy-related outcomes			X	X	X
Model related outcomes			X	X	X
Cost questionnaire					X

Data collection and management

The data underlying this study, including the outcome measures such as mortality and admission rates, will be automatically extracted from the EHR system into an electronic data capture system (Castor EDC). The questionnaires regarding patient-reported outcomes and cost-benefit analysis will subsequently be sent out digitally through the EDC system. The data will be stored for 15 years, in agreement with local regulations and with consent. Data management and monitoring will be performed by the study team. Additionally, a monitor from the local independent clinical monitoring center will monitor the data and perform data validation checks. Participant data underlying the results of this study can be shared. The data can be requested following publication of this work. The data can be shared with researchers upon reasonable request, which is allowed under local privacy regulations. Proposals should be directed to the corresponding author and requestors will need to sign a data access agreement.

Safety, adverse events, and monitoring

Adverse events will be monitored by the investigators, including device related adverse events. Serious adverse events will be reported within a maximum of 15 days to the local authority, and an annual safety report will be submitted to the local authorities. Examples of possible adverse events include delayed appropriate antibiotic

For peer review only - http://bmjopen.bmj.com/site/about/guidelines.xhtml

treatment, hospital readmission or prolonging of hospital stay. The study risk was assessed using the NFU risk classification and classified as moderate risk [14]. The study has its own data safety monitoring board (DSMB) consisting of clinicians, a statistician, and an epidemiologist, independent of the study team. The DSMB will meet before the first year of the study ends, and once a year during the study period. During the study period, the DSMB will be supplied with an interim analysis, and will advise the study team based on these results. A meeting will be held to evaluate mortality after 2850 patients have completed the trial. Additional meetings may be requested based on trial events. At least two weeks before each meeting the DSMB will receive reports regarding data quality, recruitment and patients' safety. The DSMB will report back to the principal investigator regarding any advice they may have. Additionally, the study will be monitored by an independent clinical trial monitoring committee, which performs quality checks on the data and regular on-site auditing.

Allocation and randomization

Participants will be randomly assigned to either the intervention or control group with a 1:1 allocation through a validated computer-generated random number within the EHR system. This will be a simple randomization without stratification. Randomization will occur automatically after consent and after blood cultures have been ordered. Physicians caring for a patient in the control group will not see any algorithm predictions. Physicians caring for a patient in the intervention group will be able to see the algorithm prediction. Due to the nature of the intervention, neither participant nor staff can be blinded to the allocation.

Statistical analysis

We will carry out a per-protocol analysis. The primary outcome, the 30-day mortality rate, will be compared between the groups using a non-inferiority test for the ratio of two proportions. This will be a one-sided test with a significance level of 0.05. Additionally, we will do a multivariable analysis of the primary and key secondary outcomes to adjust for potential effect of age, Charlson Comorbidity Index (CCI), use of immunosuppressive medication, Modified Early Warning Score (MEWS), and resuscitation status. Similar tests will be performed for in-hospital mortality rate and the hospital admission rate. The hospital length-of-stay will be analyzed as a continuous variable and a non-inferiority test for the differences between the two means will be used. The test statistic to be used is a one-sided T-test with a significance level of 0.05. The model-related outcomes will be assessed using an area under the curve and calibration plots. Baseline characteristics will be presented using descriptive statistics, without statistical testing for differences, since this is a randomized trial.

Patient and public involvement

Patient participation is important in both the clinical trial and the implementation of AI in clinical practice. The Client Advisory Board of Amsterdam UMC and a former sepsis patient evaluated the study question as relevant and the study design as achievable from patients' perspective. They will stay involved during the study as well. Also, to disseminate results of the study, we will collaborate with relevant patient organizations. Furthermore, a patient safety and implementation expert was involved in the development of the study protocol, and will remain

For peer review only - http://bmjopen.bmj.com/site/about/guidelines.xhtml

involved during the study period. They will continue to stay involved if the study is deemed successful and the model can be implemented into clinical practice.

Ethics and dissemination

Obtaining informed consent is of the utmost importance when working with study participants. In order to ensure that participants are well informed about the study before consenting, all potential participants will have the trial clearly explained to them by a trained member of the study team. In addition to the verbal information, a paper information brochure will be provided to each participant, see Supplementary Material 1. We inform patients about the procedures and what the consequences of doing or withholding blood cultures can be, as this is the most relevant information for them. Each participant will be made aware of their right to withdraw consent at any given time. If a patient withdraws consent their data will no longer be used in the study and the study personnel will not follow them up. If the patient was in the intervention group and blood culture analysis was already cancelled, we cannot reinstate the analysis, since the material has been destroyed. Due to the nature of the study, the potential participant will get 1 hour to consider joining the study. If any change to the protocol is made the Ethics Review Board will be notified and will have to give permission. If the changes are relevant to the participants they will receive additional information regarding the changes made.

Research results will be published in peer-reviewed journals approximately one year after the trial has ended. This will give the research team sufficient time for statistical analyses.

This study will be conducted according to the principles of the Declaration of Helsinki, and in accordance with the Medical Research Involving Human Subjects Act, General Data Privacy Regulation, and Medical Device Regulation. The study was approved by the Amsterdam University Medical Center's medical ethics review committee with number 22.0567. The trial was registered at Clinicaltrials.gov (NCT06163781).

Discussion

Blood cultures are a commonly used diagnostic tool in the ED. Unfortunately, low yields and high rates of contamination lead to unnecessary use of antibiotics, prolonged length-of-stay in the hospital, and even higher mortality. A machine learning model has been validated retrospectively and prospectively, and can identify patients at low risk for positive blood cultures. The study described in this protocol is the first randomized controlled trial using machine learning to predict blood culture outcomes. However, possible limits to this trial include restricted future application due to the exclusion of severely immunocompromised patients. Future

For peer review only - http://bmjopen.bmj.com/site/about/guidelines.xhtml

studies may need to focus on this specific subgroup as well. Nevertheless, this study will include a heterogeneous group presenting to the emergency department and may greatly influence current standards of practice. By using this model in practice, it can potentially reduce a significant amount of blood culture analyses and prevent the undesirable effects of false-positive blood culture results.

Statements

Contributors

AZ and SB contributed equally to this paper. AZ and SB wrote the first draft of the paper. AB, MS and PN conceptualized the study. AB, MS, JW and PN secured funding for the study. BLW provided the sample size calculations and statistical analysis plan. ML developed the resources. KP, RS, KA, MR, ML, JW, RdJ and PN supervised various parts of the research process within their expertise. AZ, SB, AB, MS, MR and PN revised the manuscript. All authors approved the final version of the manuscript.

Funding

This RCT is funded by ZonMW DoelmatigheidsOnderzoek, grant number 2012379. Additionally, this trial is supported by a TKI grant, which aims to use these trial results to further optimize the tool. The funders had no role in the study design, and will have no role in its execution, data analyses or decision to publish.

Competing interests

All authors have nothing to disclose.

For peer review only

For peer review only - http://bmjopen.bmj.com/site/about/guidelines.xhtml

References

1. Wang, H.E., A.R. Jones, and J.P. Donnelly, *Revised National Estimates of Emergency Department Visits for Sepsis in the United States.* Crit Care Med, 2017. **45**(9): p. 1443-1449.
2. Fabre, V., et al., *Prescribers' knowledge, attitudes and perceptions about blood culturing practices for adult hospitalized patients: a call for action.* Infect Control Hosp Epidemiol, 2018. **39**(11): p. 1394-1396.
3. Long, B. and A. Koyfman, *Best Clinical Practice: Blood Culture Utility in the Emergency Department.* J Emerg Med, 2016. **51**(5): p. 529-539.
4. Nannan Panday, R.S., et al., *Evaluation of blood culture epidemiology and efficiency in a large European teaching hospital.* PLoS One, 2019. **14**(3): p. e0214052.
5. Denny, K.J., et al., *Is it time for a culture change? Blood culture collection in the emergency department.* Emerg Med Australas, 2018. **30**(4): p. 575-577.
6. Klucher, J.M., et al., *Risk factors and clinical outcomes associated with blood culture contamination.* Infect Control Hosp Epidemiol, 2022. **43**(3): p. 291-297.
7. Doern, G.V., et al., *Practical Guidance for Clinical Microbiology Laboratories: A Comprehensive Update on the Problem of Blood Culture Contamination and a Discussion of Methods for Addressing the Problem.* Clin Microbiol Rev, 2019. **33**(1).
8. Fabre, V., et al., *Does This Patient Need Blood Cultures? A Scoping Review of Indications for Blood Cultures in Adult Nonneutropenic Inpatients.* Clin Infect Dis, 2020. **71**(5): p. 1339-1347.
9. Boerman, A.W., et al., *Using machine learning to predict blood culture outcomes in the emergency department: a single-centre, retrospective, observational study.* BMJ Open, 2022. **12**(1): p. e053332.
10. Schinkel, M., et al., *Diagnostic stewardship for blood cultures in the emergency department: A multicenter validation and prospective evaluation of a machine learning prediction tool.* EBioMedicine, 2022. **82**: p. 104176.
11. Schinkel, M., et al., *Detecting changes in the performance of a clinical machine learning tool over time.* EBioMedicine, 2023. **97**: p. 104823.
12. Herdman, M., et al., *Development and preliminary testing of the new five-level version of EQ-5D (EQ-5D-5L).* Qual Life Res, 2011. **20**(10): p. 1727-36.
13. Bouwmans, C., et al., *The iMTA Productivity Cost Questionnaire: A Standardized Instrument for Measuring and Valuing Health-Related Productivity Losses.* Value Health, 2015. **18**(6): p. 753-8.
14. (NFU), N.F.o.U.M.C., *Guideline Quality assurance of research involving human subjects*. 2023.

For peer review only - http://bmjopen.bmj.com/site/about/guidelines.xhtml

Figure Legend:

Figure 1 – SHAP values for model variables

Figure 2 – CONSORT flow chart

Figure 3 – Flow chart study design

Table 1 – Visualization study timeline

Brief explanation with Figure 4: “*not applicable to all participants

A only in intervention group when predicted probability of positive blood culture is < 5%.

B see box 2”

Box 1 – Overview of inclusion and exclusion criteria”

Brief explanation with Box 1: “* Predictions can only be made when at least 20% of the needed parameters are available for a patient, which is the case in approximately 80% of all patients who undergo blood culture analyses in the ED.”

Box 2 – Overview of outcome measures

For peer review only

For peer review only - http://bmjopen.bmj.com/site/about/guidelines.xhtml

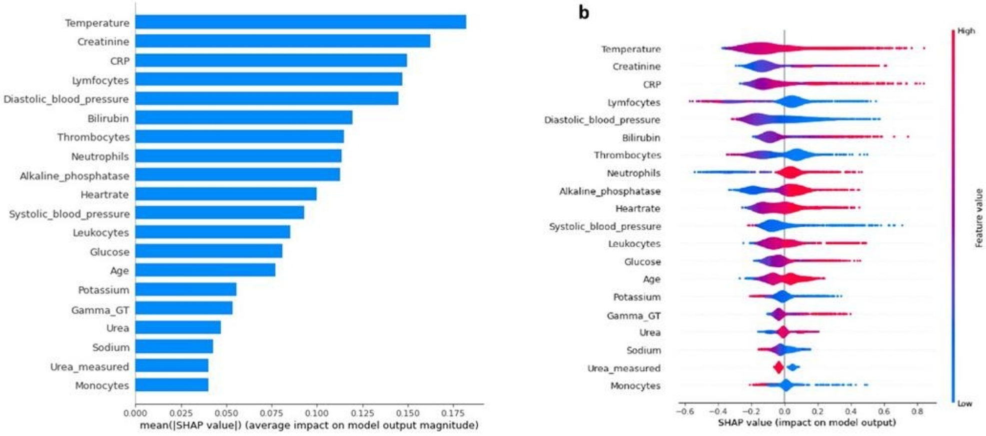

Figure 1 - SHAP values

This figure depicts SHAP (Shapley Additive Explanation) values, illustrating the individual contributions of each variable to the model's prediction. In the left panel (blue bars), we see the average impact of the 20 most important features on the prediction. Meanwhile, the right panel not only shows the feature's contribution but also its direction. Contributions to the left of 0 on the x-axis correspond to negative predictions for blood cultures, while those to the right correspond with positive predictions. Additionally, the color indicates the predictor's actual value, with blue indicating low values and red indicating high values.

277x121mm (96 x 96 DPI)

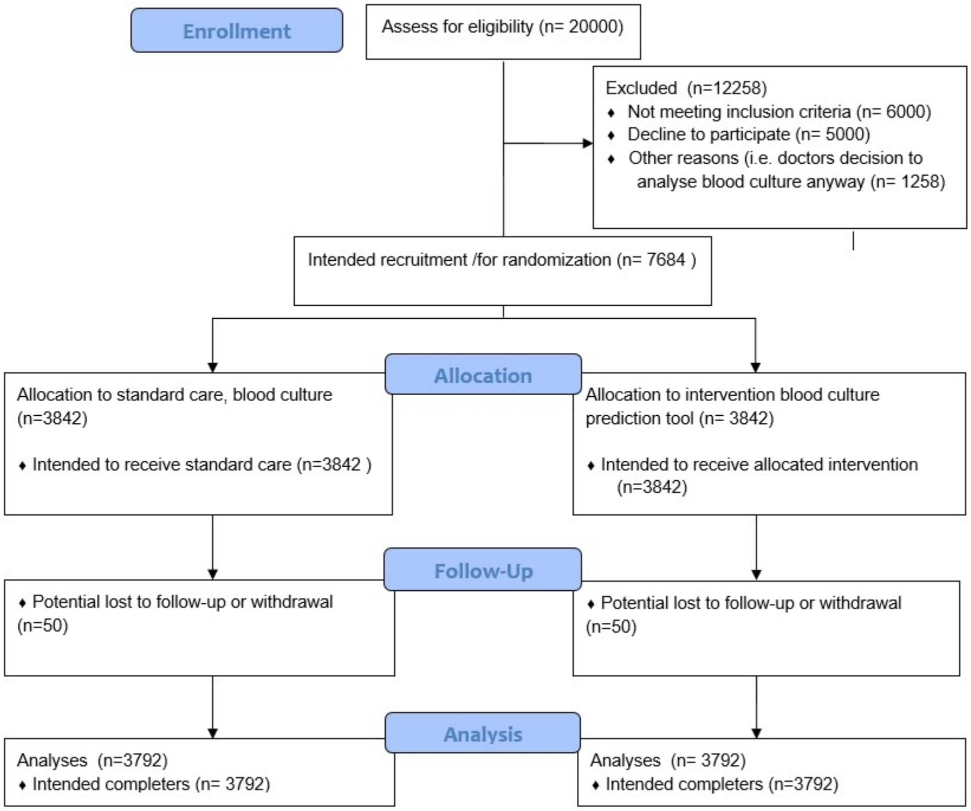

Figure 2 - CONSORT flow chart

223x184mm (96 x 96 DPI)

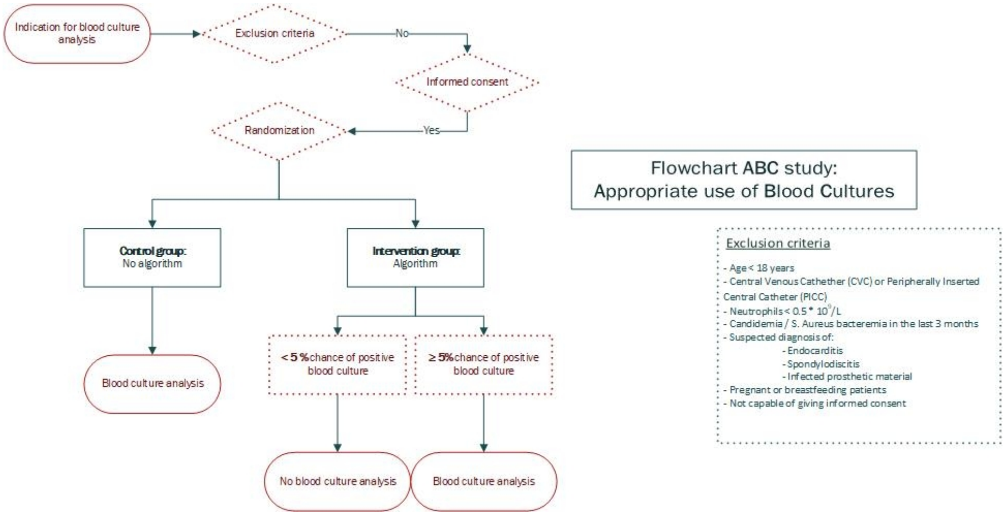

Figure 3 - Flow chart study design

255x130mm (96 x 96 DPI)

Participant Information for Participation in Medical Scientific Research

The ABC Study – Appropriate Use of Blood Cultures in the Emergency Department through Machine Learning: a Randomized Controlled Trial.

Introduction

Dear Sir/Madam,

You are receiving this letter because you have visited the emergency department of Amsterdam UMC and your attending physician intends to collect a blood culture from you, or has already done so. With this information letter, we would like to ask you to participate in medical scientific research. Participation is voluntary. You will learn about the nature of the research, its implications for you, and the advantages and disadvantages involved. It is a lot of information. Would you please read through the information and decide if you wish to participate? If you decide to participate, you can fill out the form provided in the attachment.

Ask Your Questions

You can make your decision based on the information provided in this information letter. Additionally, we recommend the following:

- Ask questions to the researcher who provided you with this information letter.

- Discuss this research with your partner, family, or friends.

- Ask questions to an independent expert

- Read information on www.rijksoverheid.nl/mensenonderzoek.

1. General Information

Amsterdam UMC – location VUmc has initiated this research. Researchers, including physicians, medical students, researchers, and research nurses, conduct the research. The Central Committee on Research Involving Human Subjects (CCMO) has validated this research, and the Medical Ethics Review Committee (METc) has approved it as well. Participation in our scientific research is voluntary. However, we require your written consent to participate.

2. What is the Purpose of the Research?

The aim of this research is to reduce unnecessary diagnostics with blood cultures, since blood cultures can have various adverse effects. Therefore, in this study, we are investigating the safety and effectiveness of a computer program that predicts the outcome of a blood culture. This prediction model assists the physician in deciding whether a blood culture should be analyzed or not. If the probability of the blood culture yielding useful results is very low, we aim to cancel the blood culture analysis and prevent potential adverse effects for the patient.

For peer review only

3. What is the Background of the Research?

Blood cultures can detect bacteria in the bloodstream. Although bloodstream infections are not common, blood cultures are frequently used as part of standard care for patients with possible infections. In the emergency department, only about 10% of blood cultures yield positive results. The other 90% provide no information or even incorrect information (a bacterium is found, but it is not the cause of the infection). Patients with such "false-positive" cultures often receive unnecessary antibiotics, undergo more procedures, and on average stay in the hospital for a longer period of time.

Our research group has developed a computer program (prediction model) that can predict the likelihood of a positive blood culture. In this study, we aim to omit blood culture analysis when the chance of a positive result is very low, thereby avoiding unnecessary testing. By analyzing fewer blood cultures, we can also reduce the number of false-positive results. Consequently, unnecessary antibiotic administration, procedures, and hospital admissions can be avoided. In this study, we aim to determine whether this approach is indeed safe and assess the benefits for patients.

For peer review only

4. How Does the Research Proceed?

How long does the research last for?

If you participate in the research, your data will be reviewed for up to 3 months after your visit to the emergency department, using the electronic patient record.

Step 1: Are You Eligible to Participate?

We first need to determine if you are eligible to participate. Therefore, the researcher checks whether your attending physician indeed intends to collect a blood culture (or has already done so). The researcher also verifies if there are sufficient data available in your patient record for the prediction model to make a prediction.

In some cases, even if the above conditions apply to you, you may still not be eligible to participate in the research. This is the case, for example, if you recently have had a bloodstream infection.

Step 2: Using the Prediction Model

For this research, we have two groups, and the allocation to these groups is determined by randomization:

• Group 1 (intervention group): The physician of the individuals in this group uses the prediction model.

• Group 2 (control group): The physician of the individuals in this group does not use the prediction model.

Only in Group 1 can the prediction model be used to determine whether the blood culture should be analyzed or not.

For peer review only - http://bmjopen.bmj.com/site/about/guidelines.xhtml

How is it different from standard care?

If you are in the control group (Group 2) of the study, there is no difference from standard care. If you are assigned to Group 1 (the intervention group), your physician may cancel the analysis of your blood culture. Normally, the physician would proceed with the analysis. However, please note that your physician will only make this decision if the prediction model indicates a very low risk for a bloodstream infection. From that point onwards, the physician will consider the blood culture as "negative" and treat you accordingly.

5. What Agreements Do We Make with You?

We want the research to proceed smoothly. Therefore, we make the following agreement with you:

- You will contact the researcher if you no longer wish to participate in the research.

For peer review only

6. What Side Effects, Adverse Effects, or Inconveniences Can You Experience?

The use of the prediction model may lead the physician to not collect a blood culture from you, whereas it would have been done otherwise. Then, there is a very small chance that a bloodstream infection may be missed. Even if such an infection is missed, it is likely to be treated effectively, or you may recover spontaneously. However, in the worst-case scenario, there is a chance of severe harm or even death due to this infection, which could have been prevented by timely treatment based on the result of a blood culture.

7. What are the Benefits and Disadvantages of Participating in the Research?

If you are allocated to the intervention group, your blood culture analysis may be canceled. This reduces unnecessary testing for you. Not only does this save costs, but it may also result in receiving fewer or different antibiotics, undergoing fewer tests, and spending less time in the hospital.

Participating in the research may also have adverse consequences, as mentioned above in point 6. Research on our prediction model has shown that the risk of these adverse consequences is very small.

Do You Not Want to Participate?

You decide whether or not to participate in the research. If you choose not to participate, you will still receive the standard care you would otherwise receive.

8. When Does the Research End?

The researcher will inform you if there is new information about the research that is important for you. The researcher will then ask you if you wish to continue participating. In the following situations, the research ends for you:

- All examinations according to the schedule are completed.
- You decide to withdraw from the research yourself. You can do this at any time. Please inform the researcher immediately. You do not need to explain why you are withdrawing.
- One of the following authorities decides that the research must stop
    - Amsterdam UMC – location VUmc,
    - The government, or the medical ethics committee that assesses the research.

For peer review only - http://bmjopen.bmj.com/site/about/guidelines.xhtml

What Happens If You Withdraw from the Research?

Inform the researcher about this. The researchers will use the data collected up to the point of withdrawal but will not collect any new data.

The entire research is concluded when the data of all participants have been checked in the medical record after 3 months.

9. What Happens After the Research?

Approximately one year after the research is completed, the researchers will publish the results of the research in a medical scientific journal.

10. What Do We Do with Your Data?

If you participate in the research, you also consent to the collection, use, and storage of your data.

What data do we store?

We store this data:

- Your gender

- Your age

- Data about your health

At the end of the research, we will also request your data once from the Personal Records Database (BRP), so that we are informed if anything happens to you outside the hospital.

Why do we collect, use, and store your data?

We collect, use, and store your data to answer the questions of this research and to publish the results.

How do we protect your privacy?

To protect your privacy, we assign a code to your data. Only this code is used on all your data. We keep the key to the code in a secure location at Amsterdam UMC. When processing your data, we always use only this code. Even in reports and publications about the research, no one can tell that it was about you.

Who can see your data?

Some people may view your name and other personal data without a code. These are people who verify whether the researchers are conducting the research properly and reliably. These people can access your data:

• An auditor working for the researcher.

• National and international supervisory authorities. For example, the Healthcare and Youth Inspectorate.

These individuals keep your data confidential. We ask you to give consent for this access.

For peer review only - http://bmjopen.bmj.com/site/about/guidelines.xhtml

For peer review only

How long do we store your data?

We store your data for 15 years in the hospital, to analyze and publish the results.

Can you withdraw your consent for the use of your data?

You can withdraw your consent for the use of your data at any time. This applies to the use in this research and in other research. However, please note: if you withdraw your consent, and researchers have already collected data for a study, they may still use this data.

Can the researchers approach you again for follow-up research?

The researchers may approach you for follow-up research. You can indicate on the consent form whether you agree to this or not.

Do You Want to Know More About Your Privacy?

- Do you want to know more about your rights regarding the processing of personal data? Then visit www.autoriteitpersoonsgegevens.nl.
- Do you have questions about your rights? Or do you have a complaint about the processing of your personal data? Then contact the person responsible for processing your personal data. For your research, that is:
    - Amsterdam UMC
- If you have complaints about the processing of your personal data, we recommend that you first discuss this with the research team. You can also contact the Data Protection Officer of Amsterdam UMC. Or you can file a complaint with the Personal Data Authority.

11. Will You Receive Compensation for Participating in the Research?

You will not receive compensation for participating in this research.

12. Are You Insured During the Research?

Insurance has been taken out for everyone participating in this research. The insurance covers damage caused by the research. But not all damage. In Appendix B, you will find more information about the insurance and the exceptions. It also states to whom you can report damage.

13. Do You Have Questions?

You can ask questions about the research to the research team. If you want advice from someone with no vested interest, consult the independent expert. They are knowledgeable about the research but are not involved in conducting it.

Do you have a complaint? Discuss this with the researcher or the physician treating you. Prefer not to do this? Then contact the hospital's complaints officer. Appendix A provides information on where to find them.

For peer review only - http://bmjopen.bmj.com/site/about/guidelines.xhtml

14. How Do You Give Consent for the Research?

You can take time to consider this research. After an hour of consideration, you inform the researcher whether you understand the information and whether you wish to participate or not. If you choose to participate, you will fill out the consent form provided with this information letter. Both you and the researcher will receive a signed version of this consent statement.

Thank you for your time.

For peer review only

For peer review only - http://bmjopen.bmj.com/site/about/guidelines.xhtml

The ABC Study – 'Appropriate use of Blood Cultures in the emergency department through machine learning: a randomized controlled trial.'

I have read the participant information associated with the ABC study. I understand the information provided. My questions have been answered satisfactorily, and I had sufficient time to decide whether or not to participate in this study. I understand that my participation is entirely voluntary. I am aware that I can withdraw at any time without giving a reason.

• I consent to the use, storage, and processing of my encoded data for research purposes related to blood cultures.

• I consent to the retention of my encoded data for 15 years.

• I understand that for the monitoring of the research, some individuals may access all of my data. These individuals are listed in this information letter. I authorize these individuals to access my data for monitoring purposes.

Please indicate below yes or no:

I consent to potentially being asked to participate in a follow-up study after this research.

Yes ☐ No ☐

I consent to the retrieval of my data from the Personal Records Database (BRP).
Yes ☐ No ☐

Name: _______________________ Signature: _________________

Date: _______________________

I hereby declare that I have fully informed this participant about the ABC study. If any information arises during the study that may affect the participant's consent, I will inform them promptly.

Researcher's Name (or representative): _______________________

Signature: _________________

Date: _______________________

For peer review only - http://bmjopen.bmj.com/site/about/guidelines.xhtml

SPIRIT

STANDARD PROTOCOL ITEMS: RECOMMENDATIONS FOR INTERVENTIONAL TRIALS

SPIRIT 2013 Checklist: Recommended items to address in a clinical trial protocol and related documents*

Section/item	Item No	Description	Addressed on page number
**Administrative information**			
Title	1	Descriptive title identifying the study design, population, interventions, and, if applicable, trial acronym	_____1________
Trial registration	2a	Trial identifier and registry name. If not yet registered, name of intended registry	_____2________
	2b	All items from the World Health Organization Trial Registration Data Set	On Clinical Trials site
Protocol version	3	Date and version identifier	In original protocol file
Funding	4	Sources and types of financial, material, and other support	______9______
Roles and responsibilities	5a	Names, affiliations, and roles of protocol contributors	______9______
	5b	Name and contact information for the trial sponsor	_____1_______
	5c	Role of study sponsor and funders, if any, in study design; collection, management, analysis, and interpretation of data; writing of the report; and the decision to submit the report for publication, including whether they will have ultimate authority over any of these activities	_______9_____

For peer review only

	5d	Composition, roles, and responsibilities of the coordinating centre, steering committee, endpoint adjudication committee, data management team, and other individuals or groups overseeing the trial, if applicable (see Item 21a for data monitoring committee)	In original protocol
**Introduction**			
Background and rationale	6a	Description of research question and justification for undertaking the trial, including summary of relevant studies (published and unpublished) examining benefits and harms for each intervention	_____3,4,5____
	6b	Explanation for choice of comparators	______5______
Objectives	7	Specific objectives or hypotheses	______3,5______
Trial design	8	Description of trial design including type of trial (eg, parallel group, crossover, factorial, single group), allocation ratio, and framework (eg, superiority, equivalence, noninferiority, exploratory)	______3,4______
**Methods: Participants, interventions, and outcomes**			
Study setting	9	Description of study settings (eg, community clinic, academic hospital) and list of countries where data will be collected. Reference to where list of study sites can be obtained	______4_______
Eligibility criteria	10	Inclusion and exclusion criteria for participants. If applicable, eligibility criteria for study centres and individuals who will perform the interventions (eg, surgeons, psychotherapists)	______4______
Interventions	11a	Interventions for each group with sufficient detail to allow replication, including how and when they will be administered	______4_____
	11b	Criteria for discontinuing or modifying allocated interventions for a given trial participant (eg, drug dose change in response to harms, participant request, or improving/worsening disease)	Not applicable
	11c	Strategies to improve adherence to intervention protocols, and any procedures for monitoring adherence (eg, drug tablet return, laboratory tests)	Not applicable
	11d	Relevant concomitant care and interventions that are permitted or prohibited during the trial	Not applicable

For peer review only - http://bmjopen.bmj.com/site/about/guidelines.xhtml

Outcomes	12	Primary, secondary, and other outcomes, including the specific measurement variable (eg, systolic blood pressure), analysis metric (eg, change from baseline, final value, time to event), method of aggregation (eg, median, proportion), and time point for each outcome. Explanation of the clinical relevance of chosen efficacy and harm outcomes is strongly recommended	________5_____
Participant timeline	13	Time schedule of enrolment, interventions (including any run-ins and washouts), assessments, and visits for participants. A schematic diagram is highly recommended (see Figure)	______13_____
Sample size	14	Estimated number of participants needed to achieve study objectives and how it was determined, including clinical and statistical assumptions supporting any sample size calculations	________5_____
Recruitment	15	Strategies for achieving adequate participant enrolment to reach target sample size	________5_____

Methods: Assignment of interventions (for controlled trials)

Allocation:

Sequence generation	16a	Method of generating the allocation sequence (eg, computer-generated random numbers), and list of any factors for stratification. To reduce predictability of a random sequence, details of any planned restriction (eg, blocking) should be provided in a separate document that is unavailable to those who enrol participants or assign interventions	_____6______
Allocation concealment mechanism	16b	Mechanism of implementing the allocation sequence (eg, central telephone; sequentially numbered, opaque, sealed envelopes), describing any steps to conceal the sequence until interventions are assigned	_____6_______
Implementation	16c	Who will generate the allocation sequence, who will enrol participants, and who will assign participants to interventions	_____6_______
Blinding (masking)	17a	Who will be blinded after assignment to interventions (eg, trial participants, care providers, outcome assessors, data analysts), and how	_____6_______
	17b	If blinded, circumstances under which unblinding is permissible, and procedure for revealing a participant’s allocated intervention during the trial	Not applicable

Methods: Data collection, management, and analysis

Data collection methods	18a	Plans for assessment and collection of outcome, baseline, and other trial data, including any related processes to promote data quality (eg, duplicate measurements, training of assessors) and a description of study instruments (eg, questionnaires, laboratory tests) along with their reliability and validity, if known. Reference to where data collection forms can be found, if not in the protocol	______5______
	18b	Plans to promote participant retention and complete follow-up, including list of any outcome data to be collected for participants who discontinue or deviate from intervention protocols	Not applicable
Data management	19	Plans for data entry, coding, security, and storage, including any related processes to promote data quality (eg, double data entry; range checks for data values). Reference to where details of data management procedures can be found, if not in the protocol	______5______
Statistical methods	20a	Statistical methods for analysing primary and secondary outcomes. Reference to where other details of the statistical analysis plan can be found, if not in the protocol	______5,6_____
	20b	Methods for any additional analyses (eg, subgroup and adjusted analyses)	______6______
	20c	Definition of analysis population relating to protocol non-adherence (eg, as randomised analysis), and any statistical methods to handle missing data (eg, multiple imputation)	______6______
**Methods: Monitoring**			
Data monitoring	21a	Composition of data monitoring committee (DMC); summary of its role and reporting structure; statement of whether it is independent from the sponsor and competing interests; and reference to where further details about its charter can be found, if not in the protocol. Alternatively, an explanation of why a DMC is not needed	______6______
	21b	Description of any interim analyses and stopping guidelines, including who will have access to these interim results and make the final decision to terminate the trial	______6______
Harms	22	Plans for collecting, assessing, reporting, and managing solicited and spontaneously reported adverse events and other unintended effects of trial interventions or trial conduct	_____5,6______
Auditing	23	Frequency and procedures for auditing trial conduct, if any, and whether the process will be independent from investigators and the sponsor	______6______
**Ethics and dissemination**			

Research ethics approval	24	Plans for seeking research ethics committee/institutional review board (REC/IRB) approval	______7______
Protocol amendments	25	Plans for communicating important protocol modifications (eg, changes to eligibility criteria, outcomes, analyses) to relevant parties (eg, investigators, REC/IRBs, trial participants, trial registries, journals, regulators)	______7______
Consent or assent	26a	Who will obtain informed consent or assent from potential trial participants or authorised surrogates, and how (see Item 32)	______7______
	26b	Additional consent provisions for collection and use of participant data and biological specimens in ancillary studies, if applicable	Not applicable
Confidentiality	27	How personal information about potential and enrolled participants will be collected, shared, and maintained in order to protect confidentiality before, during, and after the trial	______5______
Declaration of interests	28	Financial and other competing interests for principal investigators for the overall trial and each study site	______9______
Access to data	29	Statement of who will have access to the final trial dataset, and disclosure of contractual agreements that limit such access for investigators	______5______
Ancillary and post-trial care	30	Provisions, if any, for ancillary and post-trial care, and for compensation to those who suffer harm from trial participation	Supplement 1
Dissemination policy	31a	Plans for investigators and sponsor to communicate trial results to participants, healthcare professionals, the public, and other relevant groups (eg, via publication, reporting in results databases, or other data sharing arrangements), including any publication restrictions	_____7_______
	31b	Authorship eligibility guidelines and any intended use of professional writers	Not applicable
	31c	Plans, if any, for granting public access to the full protocol, participant-level dataset, and statistical code	______5______
**Appendices**			
Informed consent materials	32	Model consent form and other related documentation given to participants and authorised surrogates	Supplement 1

For peer review only - http://bmjopen.bmj.com/site/about/guidelines.xhtml

Biological specimens	33	Plans for collection, laboratory evaluation, and storage of biological specimens for genetic or molecular analysis in the current trial and for future use in ancillary studies, if applicable	Not applicable

*It is strongly recommended that this checklist be read in conjunction with the SPIRIT 2013 Explanation & Elaboration for important clarification on the items. Amendments to the protocol should be tracked and dated. The SPIRIT checklist is copyrighted by the SPIRIT Group under the Creative Commons "Attribution-NonCommercial-NoDerivs 3.0 Unported" license.

For peer review only

BMJ Open

Appropriate use of Blood Cultures in the emergency department through machine learning (ABC): study protocol for a randomized controlled non-inferiority trial

Journal:	*BMJ Open*
Manuscript ID	bmjopen-2024-084053.R2
Article Type:	Protocol
Date Submitted by the Author:	09-May-2024
Complete List of Authors:	van der Zaag, Anuschka; Amsterdam UMC Locatie VUmc, Department of Internal Medicine, Division of Acute Medicine<br>Bhagirath, Sheena C.; Amsterdam UMC Locatie VUmc, Department of Internal Medicine, Division of Acute Medicine<br>Boerman, Anneroos; Amsterdam UMC Locatie VUmc, Department of Internal Medicine, Division of Acute Medicine; Amsterdam UMC Locatie VUmc, Department of Laboratory Medicine<br>Schinkel, Michiel; Amsterdam University Medical Centres, Center for Experimental and Molecular Medicine (C.E.M.M.); Amsterdam UMC Locatie VUmc, Department of Internal Medicine, Division of Acute Medicine<br>Paranjape, Ketan ; Amsterdam UMC Locatie VUmc, Department of Internal Medicine, Division of Acute Medicine<br>Azijli, Kaoutar; Amsterdam UMC Locatie VUmc, Department of Emergency Medicine<br>Ridderikhof, Milan L. ; Amsterdam UMC Locatie AMC, Department of Emergency Medicine<br>Lie, Mei; Amsterdam UMC Locatie VUmc, Department of EVA Service Center<br>Lissenberg-Witte, Birgit; Amsterdam UMC Locatie VUmc, Department of Epidemiology & Data Science<br>Schade, Rogier; Amsterdam UMC Locatie VUmc, Department of Medical Microbiology and Infection Prevention<br>Wiersinga, Joost; Amsterdam UMC Locatie AMC, Department of Internal Medicine, Division of Infectious Diseases; Amsterdam UMC Locatie AMC, Center for Experimental and Molecular Medicine (C.E.M.M.)<br>de Jonge, Robert; Amsterdam UMC Locatie VUmc, Department of Laboratory Medicine<br>Nanayakkara, Prabath; Amsterdam UMC Locatie VUmc, Department of Internal Medicine, Section General Internal Medicine
**Primary Subject Heading**:	Emergency medicine
Secondary Subject Heading:	Infectious diseases, Health informatics
Keywords:	ACCIDENT & EMERGENCY MEDICINE, Clinical Decision-Making, INFECTIOUS DISEASES, Diagnostic microbiology < INFECTIOUS DISEASES, INTERNAL MEDICINE

SCHOLARONE™
Manuscripts

Appropriate use of Blood Cultures in the emergency department through machine learning (ABC): study protocol for a randomized controlled non-inferiority trial

Anuschka Y. van der Zaag[1], Sheena C. Bhagirath[1*], Anneroos W. Boerman[1,2], Michiel Schinkel[1,3], Ketan Paranjape[1], Kaoutar Azijli[4], Milan L. Ridderikhof[4], Mei Lie[5], Birgit Lissenberg-Witte[6], Rogier Schade[7], W. Joost Wiersinga[3,8], Robert de Jonge[2], Prabath W.B. Nanayakkara[1]*

[*]These authors contributed equally to this paper.

[1]Department of Internal Medicine, Section General Internal Medicine, Amsterdam UMC, VU University, Amsterdam, Netherlands

[2]Department of Laboratory Medicine, Amsterdam UMC, Amsterdam, Netherlands

[3]Center for Experimental and Molecular Medicine (CEMM), Amsterdam UMC, University of Amsterdam, Amsterdam, Netherlands

[4]Department of Emergency Medicine, Amsterdam UMC, Amsterdam, Netherlands

[5]Department of EVA Service Center, Amsterdam UMC, Amsterdam, Netherlands

[6]Department of Epidemiology & Data Science, Amsterdam UMC, Netherlands

[7]Department of Medical Microbiology and Infection Prevention, Amsterdam UMC, Amsterdam, Netherlands

[8]Department of Internal Medicine, Division of Infectious Diseases, Amsterdam UMC, University of Amsterdam, Amsterdam, Netherlands

Correspondence to:

Prabath WB Nanayakkara

Section General Internal Medicine, Department of Internal Medicine, Amsterdam UMC location VUmc, De Boelelaan 1118, Amsterdam, 1081 HZ, Netherlands

Email: p.nanayakkara@amsterdamumc.nl

For peer review only - http://bmjopen.bmj.com/site/about/guidelines.xhtml

Abstract

Introduction

The liberal use of blood cultures in emergency departments leads to low yields and high numbers of false-positive results. False-positive, contaminated cultures are associated with prolonged hospital stays, increased antibiotic usage, and even higher hospital mortality rates. This trial aims to investigate whether a recently developed and validated machine learning model for predicting blood culture outcomes can safely and effectively guide clinicians in withholding unnecessary blood culture analysis.

Methods and analysis

A randomized controlled, non-inferiority trial comparing current practice with a machine learning guided approach. The primary objective is to determine whether the machine learning based approach is non-inferior to standard practice based on 30-day mortality. Secondary outcomes include hospital length-of stay and hospital admission rates. Other outcomes include model performance and antibiotic usage. Participants will be recruited in the emergency departments of multiple hospitals in the Netherlands. A total of 7584 participants will be included.

Ethics and dissemination

Possible participants will receive verbal information and a paper information brochure regarding the trial. They will be given at least one hour consideration time before providing informed consent. Research results will be published in peer-reviewed journals. This study has been approved by the Amsterdam University Medical Centers' local medical ethics review committee (nr. 22.0567). The study will be conducted in concordance with the principles of the Declaration of Helsinki, and in accordance with the Medical Research Involving Human Subjects Act, General Data Privacy Regulation, and Medical Device Regulation.

Trial registration

ClinicalTrials.gov, NCT06163781.

For peer review only

Strengths and limitations of this study

- Pioneering the use of machine learning in a randomized controlled trial to predict blood culture outcomes.
- Conducting a comparative analysis against current clinical practices.
- Assessing patient-centered outcomes and performing an economic evaluation.

For peer review only - http://bmjopen.bmj.com/site/about/guidelines.xhtml

- May restrict future applications due to the exclusion of immunocompromised patients.

INTRODUCTION

Over 20% of adult emergency department visits occur due to infections [1]. Often, physicians will request blood cultures in these patients due to fear of a blood stream infection (BSI) [2]. Because of the liberal use of blood cultures, the yield tends to be low. Depending on the setting, only about 1-15% of blood cultures grow pathogens, of which 30-55% turn out to be false-positive, contaminated results [3-5]. Those contaminated cultures are associated with prolonged hospital stays, unnecessary use of antibiotics, and even hospital mortality [3, 6, 7]. To optimize blood culture use, it is crucial to better target which patients would benefit from blood culture analysis [8].

A machine learning model based on extreme gradient-boosting (XG Boost) that can predict the outcome of blood cultures in the emergency department has been developed to address this clinical dilemma, aiming to reduce unnecessary tests in low-risk patients, and help avoid the harms associated with false-positive results due to blood culture contamination [9, 10]. The performance of the model has been internally and externally validated in various settings, including a real-time background evaluation in the Amsterdam University Medical Centers (Amsterdam UMC, locations VUmc and AMC) Electronic Health Records (EHR) system, where it maintained an Area Under the Operating Curve (AUC) of 0.76 and showed a potential reduction of approximately 30% blood cultures in the future [9, 10]. Additionally, the model has shown a consistent performance over time despite changes in clinical practice and patient characteristics [11].

The aim of this study is to investigate the safety and potential clinical benefits of using the blood culture prediction tool as decision support in reducing unnecessary blood cultures and thereby the negative side effects of false positive results for patients. This will be the first randomized controlled trial testing such a machine learning prediction tool in clinical practice.

For peer review only

METHODS AND ANALYSIS

This study protocol adheres to the Standard Protocol Items: Recommendations for Interventional Trials (SPIRIT) statement.

Model development and validation

This segment features a summary of the model development, more detailed descriptions have been published previously [9, 10]. For the development of the model data from the VUmc was split into a training (80%) and a test set (20%), stratified by blood culture outcomes. Missing data was imputed by medians. The model was trained on the training set and subsequently validated in the VUmc test set, and three other hospital datasets (AMC), Zaans Medisch Centrum (ZMC, Zaandam, Netherlands) and Beth Israel Deaconess Medical Center (BIDMC, Boston, MA, USA). These hospitals differ in size and population, comprising two tertiary teaching hospitals and one secondary care hospital in the Netherlands, along with one hospital dataset from the United States. Part of the data was gathered during the COVID-19 pandemic, but this did not affect its performance. The model contains 49 variables, namely age, sex, six vital sign measurements, and eighteen laboratory tests. Additionally, indicator variables of the laboratory tests were included. Figure 1 shows the 20 most important variables. The Area Under the Receiver Operating Characteristic (AUROC) Curves in the various validation cohorts ranged between 0.75-0.81. A prospective validation was also performed, integrating the model into the EHR of VUmc, showing an AUROC of 0.76. With the threshold for blood culture analysis set at 5%, analyses could be avoided in approximately 30%.

Study design

This study will be a multi-center randomized controlled non-inferiority trial. Participants will be randomized into an intervention and control arm. In the intervention group, the blood culture prediction model is used to predict the probability of a positive blood culture in the patient. The model will start predicting when sufficient variables are available in the EHR. A new prediction will be made every 20 minutes until 3 hours have passed since check—in of the patient in the ED. The predictions made by the model will be shown to the physician after randomization in a dashboard integrated in the electronic health records (EHR). The blood culture analysis will be canceled if the probability of a positive culture is less than 5%. This will be done manually by the study team. If the probability is over or equal to 5%, the blood culture will be analyzed as per standard practice. In the control group, all patients will have their blood cultures analyzed. The study design is summarized in Figure 2 and Figure 3. Patients will be randomized within the EHR system, to ensure that physicians cannot be influenced in their treatment decisions by seeing the score before the patient is included. The first patient was included in February 2024. Follow-up of the last patients is expected in February 2027.

Population

All adult patients (aged 18 years or older) presenting to the emergency departments (ED) of multiple hospitals in the Netherlands, and in whom blood cultures are ordered during ED stay will be assessed for eligibility. This process is automated within the EHR. The treating physician determines whether there is an indication for

For peer review only - http://bmjopen.bmj.com/site/about/guidelines.xhtml

performing blood cultures according to the current local standards, after which both the patient and study team are informed about possible study recruitment. Inclusion and exclusion criteria are summarized in Box 1. Some patients will be excluded due to the higher possibility of BSIs with a pathogen generally considered a contaminant (e.g. a central line in situ) or because of the possibility of severe clinical implications of omitting blood cultures (e.g. severe neutropenia). Since the study's goal is to reduce unnecessary testing, patients for whom blood culture analysis is deemed imperative are excluded (e.g. patients with a suspected diagnosis of endocarditis, spondylodiscitis or infected prosthetic material). Furthermore, patients unable to give informed consent and pregnant or breastfeeding patients will be excluded, due to them being considered high-risk groups by the ethics review board. Therefore, the review board did not give permission to include these groups.

**Box 1. Overview of inclusion and exclusion criteria**	
**Inclusion criteria**	Age ≥ 18 years
	Willing and able to give informed consent to participate
	Have a clinical indication for blood culture analysis (according to the treating physician)
	Have sufficient data recorded (laboratory results and vital sign measurements) for a prediction to be made*
**Exclusion criteria**	Patients with a Central Venous Catheter (CVC) or Peripherally Inserted Central Catheter (PICC) in situ
	Neutrophil count < 0.5 * 109/L
	Candidemia or S. aureus bacteremia in the past 3 months
	Suspected endocarditis, spondylodiscitis or infected prosthetic material
	Pregnant or breastfeeding patients
	Inability to give informed consent

*Predictions can only be made when at least 20% of the needed parameters are available for a patient, which is the case in approximately 80% of all patients who undergo blood culture analyses in the ED.

Sample size calculation

An analysis of 5907 unique ED visits with blood culture sampling in Amsterdam UMC showed a 30-day mortality rate of 7.6%, a hospital admission rate of 67.4%, and an average length of stay in the hospital of 6.7 days. Based on these numbers, the sample sizes with a relative non-inferiority margin of 1.25 for the rates and 1 day for the

length of stay were calculated. The sample sizes were calculated by a statistician based on a one-sided alpha of 5% and a target power of at least 80%. For 30-day mortality, the calculated sample size was 6066 (3033 per arm) to test non-inferiority. After inflation for a dropout rate of 20%, the final sample size is 7584 (3792 per arm). For the secondary outcomes, described in Box 2, fewer participants were needed for adequate power.

Recruitment

In order to achieve adequate participant enrolment, an announcement regarding the study is built into the EHR system. When the physician initiates a blood culture request in the EHR for an adult patient in the ED, a pop-up notification will automatically appear. This pop-up briefly outlines the study and presents both inclusion and exclusion criteria. If the patient provides permission to receive information about the study, the study team will be alerted and will provide the patient with additional information and obtain informed consent.

Outcomes

The primary objective is to investigate whether using a machine learning based blood culture prediction tool is non-inferior to current practice regarding 30-day all-cause mortality. All secondary outcomes are described in Box 2. Model performance will be evaluated using the AUROC, AUPRC and calibration for the model in the complete study population and in subgroups based on comorbidities. This will be assessed every three months so model performance over time can be guaranteed. The model will also continue to undergo prospective validation in the background of the EHR. Additionally, patient reported outcomes will be included to determine if the model also influences patient-reported outcome measures such as side-effects from antibiotics and quality of life. For assessment of quality of life the EQ-5D-5L questionnaire [12] will be filled out at baseline and at 3 months. Lastly, a cost-effectiveness analysis will be performed using cost questionnaires based on the iMCQ and iPCQ [13]. A timeline visualizing data collection is shown in Table 1.

For peer review

**Box 2. Overview of outcome measures**	
**Primary outcome**	30-day all cause mortality
**Key secondary outcomes**	In-hospital mortality
	Hospital admission rates
	Hospital length-of-stay
**Other outcome measures**	
**Patient-related outcomes**	90-day mortality
	30-day readmission rates
	Emergency department length-of-stay in hours
**Diagnostics related outcomes**	Percentage of blood cultures avoided in the intervention group
	Number of blood cultures on each day of hospital stay (in admitted patients)
	Percentage of positive blood cultures in each group
	Total number of laboratory- and microbiology tests in the ED
	Total number of laboratory- and microbiology test on each day of hospital stay (in admitted patients)
**Therapy-related outcomes**	Percentage of patients receiving antibiotics in the ED
	Duration of antibiotic therapy
	Types of antibiotics given in the ED

For peer review only - http://bmjopen.bmj.com/site/about/guidelines.xhtml

**Model-related outcomes**	Model performance (AUC) during the trial
	Model performance in subgroups:<br>• Immunocompromised patients (triple immunosuppressive therapy)<br>• Transplanted patients

Table 1. Data collection timeline

Allocation	Enrol ment	Allocation	Baseline	During admission*	3 months after baseline
Eligibility screen	X				
Informed consent	X				
Allocation		X			
Vital parameters			X		
Lab values			X		
Quality of life (according to EQ-5D-5L)			X		X
Canceling blood culture[A]			X[A]		
Admission rates			X		
Mortality				X	X
Patient-related outcomes[B]			X	X	X
Diagnostics related outcomes			X	X	X
Therapy-related outcomes			X	X	X
Model related outcomes			X	X	X
Cost questionnaire					X

*Not applicable to all participants. A: Only in intervention group when predicted probability of positive blood culture is < 5%. B: See Box 2.

Data collection and management

The data underlying this study, including the outcome measures such as mortality and admission rates, will be automatically extracted from the EHR system into an electronic data capture system (Castor EDC). The questionnaires regarding patient-reported outcomes and cost-benefit analysis will subsequently be sent out digitally through the EDC system. The data will be stored for 15 years, in agreement with local regulations and with consent. Data management and monitoring will be performed by the study team. Additionally, a monitor from the local independent clinical monitoring center will monitor the data and perform data validation checks. Participant data underlying the results of this study can be shared. The data can be requested following publication of this work. The data can be shared with researchers upon reasonable request, which is allowed

For peer review only - http://bmjopen.bmj.com/site/about/guidelines.xhtml

under local privacy regulations. Proposals should be directed to the corresponding author and requestors will need to sign a data access agreement.

Safety, adverse events, and monitoring

Adverse events will be monitored by the investigators, including device related adverse events. Serious adverse events will be reported within a maximum of 15 days to the local authority, and an annual safety report will be submitted to the local authorities. Examples of possible adverse events include delayed appropriate antibiotic treatment, hospital readmission or prolonging of hospital stay. The study risk was assessed using the NFU risk classification and classified as moderate risk [14]. The study has its own data safety monitoring board (DSMB) consisting of clinicians, a statistician, and an epidemiologist, independent of the study team. The DSMB will meet before the first year of the study ends, and once a year during the study period. During the study period, the DSMB will be supplied with an interim analysis, and will advise the study team based on these results. A meeting will be held to evaluate mortality after 2850 patients have completed the trial. Additional meetings may be requested based on trial events. At least two weeks before each meeting the DSMB will receive reports regarding data quality, recruitment and patients' safety. The DSMB will report back to the principal investigator regarding any advice they may have. Additionally, the study will be monitored by an independent clinical trial monitoring committee, which performs quality checks on the data and regular on-site auditing.

Allocation and randomization

Participants will be randomly assigned to either the intervention or control group with a 1:1 allocation through a validated computer-generated random number within the EHR system. This will be a simple randomization without stratification. Randomization will occur automatically after consent and after blood cultures have been ordered. Physicians caring for a patient in the control group will not see any algorithm predictions. Physicians caring for a patient in the intervention group will be able to see the algorithm prediction. Due to the nature of the intervention, neither participant nor staff can be blinded to the allocation.

Statistical analysis

We will carry out a per-protocol analysis. The primary outcome, the 30-day mortality rate, will be compared between the groups using a non-inferiority test for the ratio of two proportions. This will be a one-sided test with a significance level of 0.05. Additionally, we will do a multivariable analysis of the primary and key secondary outcomes to adjust for potential effect of age, Charlson Comorbidity Index (CCI), use of immunosuppressive medication, Modified Early Warning Score (MEWS), and resuscitation status. Similar tests will be performed for in-hospital mortality rate and the hospital admission rate. The hospital length-of-stay will be analyzed as a continuous variable and a non-inferiority test for the differences between the two means will be used. The test statistic to be used is a one-sided T-test with a significance level of 0.05. The model-related outcomes will be assessed using an area under the curve and calibration plots. Baseline characteristics will be presented using descriptive statistics, without statistical testing for differences, since this is a randomized trial.

For peer review only - http://bmjopen.bmj.com/site/about/guidelines.xhtml

For peer review only

Patient and public involvement

Patient participation is important in both the clinical trial and the implementation of AI in clinical practice. The Client Advisory Board of Amsterdam UMC and a former sepsis patient evaluated the study question as relevant and the study design as achievable from patients' perspective. They will stay involved during the study as well. Also, to disseminate results of the study, we will collaborate with relevant patient organizations. Furthermore, a patient safety and implementation expert was involved in the development of the study protocol, and will remain involved during the study period. They will continue to stay involved if the study is deemed successful and the model can be implemented into clinical practice.

ETHICS AND DISSEMINATION

Obtaining informed consent is of the utmost importance when working with study participants. In order to ensure that participants are well informed about the study before consenting, all potential participants will have the trial clearly explained to them by a trained member of the study team. In addition to the verbal information, a paper information brochure will be provided to each participant, see Supplementary Material 1. We inform patients about the procedures and what the consequences of doing or withholding blood cultures can be, as this is the most relevant information for them. Each participant will be made aware of their right to withdraw consent at any given time. If a patient withdraws consent their data will no longer be used in the study and the study personnel will not follow them up. If the patient was in the intervention group and blood culture analysis was already cancelled, we cannot reinstate the analysis, since the material has been destroyed. Due to the nature of the study, the potential participant will get 1 hour to consider joining the study. If any change to the protocol is made the Ethics Review Board will be notified and will have to give permission. If the changes are relevant to the participants, they will receive additional information regarding the changes made.

Research results will be published in peer-reviewed journals approximately one year after the trial has ended. This will give the research team sufficient time for statistical analyses.

This study will be conducted according to the principles of the Declaration of Helsinki, and in accordance with the Medical Research Involving Human Subjects Act, General Data Privacy Regulation, and Medical Device Regulation. The study was approved by the Amsterdam University Medical Center's medical ethics review committee with number 22.0567. The trial was registered at Clinicaltrials.gov (NCT06163781).

For peer review only - http://bmjopen.bmj.com/site/about/guidelines.xhtml

DISCUSSION

Blood cultures are a commonly used diagnostic tool in the ED. Unfortunately, low yields and high rates of contamination lead to unnecessary use of antibiotics, prolonged length-of-stay in the hospital, and even higher mortality. A machine learning model has been validated retrospectively and prospectively, and can identify patients at low risk for positive blood cultures. The study described in this protocol is the first randomized controlled trial using machine learning to predict blood culture outcomes. However, possible limits to this trial include restricted future application due to the exclusion of severely immunocompromised patients. Future studies may need to focus on this specific subgroup as well. Nevertheless, this study will include a heterogeneous group presenting to the emergency department and may greatly influence current standards of practice. By using this model in practice, it can potentially reduce a significant amount of blood culture analyses and prevent the undesirable effects of false-positive blood culture results.

Statements

Contributors

AZ and SB contributed equally to this paper. AZ and SB wrote the first draft of the paper. AB, MS and PN conceptualized the study. AB, MS, JW and PN secured funding for the study. BLW provided the sample size calculations and statistical analysis plan. ML developed the resources. KP, RS, KA, MR, ML, JW, RdJ and PN supervised various parts of the research process within their expertise. AZ, SB, AB, MS, MR and PN revised the manuscript. All authors approved the final version of the manuscript.

Funding

This RCT is funded by ZonMW DoelmatigheidsOnderzoek, grant number 2012379. Additionally, this trial is supported by a TKI grant, which aims to use these trial results to further optimize the tool. The funders had no role in the study design, and will have no role in its execution, data analyses or decision to publish.

Competing interests

All authors have nothing to disclose.

For peer review only

References

1. Wang, H.E., A.R. Jones, and J.P. Donnelly, *Revised National Estimates of Emergency Department Visits for Sepsis in the United States.* Crit Care Med, 2017. **45**(9): p. 1443-1449.
2. Fabre, V., et al., *Prescribers' knowledge, attitudes and perceptions about blood culturing practices for adult hospitalized patients: a call for action.* Infect Control Hosp Epidemiol, 2018. **39**(11): p. 1394-1396.
3. Long, B. and A. Koyfman, *Best Clinical Practice: Blood Culture Utility in the Emergency Department.* J Emerg Med, 2016. **51**(5): p. 529-539.
4. Nannan Panday, R.S., et al., *Evaluation of blood culture epidemiology and efficiency in a large European teaching hospital.* PLoS One, 2019. **14**(3): p. e0214052.
5. Denny, K.J., et al., *Is it time for a culture change? Blood culture collection in the emergency department.* Emerg Med Australas, 2018. **30**(4): p. 575-577.
6. Klucher, J.M., et al., *Risk factors and clinical outcomes associated with blood culture contamination.* Infect Control Hosp Epidemiol, 2022. **43**(3): p. 291-297.
7. Doern, G.V., et al., *Practical Guidance for Clinical Microbiology Laboratories: A Comprehensive Update on the Problem of Blood Culture Contamination and a Discussion of Methods for Addressing the Problem.* Clin Microbiol Rev, 2019. **33**(1).
8. Fabre, V., et al., *Does This Patient Need Blood Cultures? A Scoping Review of Indications for Blood Cultures in Adult Nonneutropenic Inpatients.* Clin Infect Dis, 2020. **71**(5): p. 1339-1347.
9. Boerman, A.W., et al., *Using machine learning to predict blood culture outcomes in the emergency department: a single-centre, retrospective, observational study.* BMJ Open, 2022. **12**(1): p. e053332.
10. Schinkel, M., et al., *Diagnostic stewardship for blood cultures in the emergency department: A multicenter validation and prospective evaluation of a machine learning prediction tool.* EBioMedicine, 2022. **82**: p. 104176.
11. Schinkel, M., et al., *Detecting changes in the performance of a clinical machine learning tool over time.* EBioMedicine, 2023. **97**: p. 104823.
12. Herdman, M., et al., *Development and preliminary testing of the new five-level version of EQ-5D (EQ-5D-5L).* Qual Life Res, 2011. **20**(10): p. 1727-36.
13. Bouwmans, C., et al., *The iMTA Productivity Cost Questionnaire: A Standardized Instrument for Measuring and Valuing Health-Related Productivity Losses.* Value Health, 2015. **18**(6): p. 753-8.
14. (NFU), N.F.o.U.M.C., *Guideline Quality assurance of research involving human subjects*. 2023.

For peer review only

For peer review only - http://bmjopen.bmj.com/site/about/guidelines.xhtml

302

303 **Figure titles:**

304 **Figure 1.** SHAP values for model variables

305 **Figure 2.** CONSORT flowchart

306 **Figure 3.** Study design flowchart

For peer review only

For peer review only - http://bmjopen.bmj.com/site/about/guidelines.xhtml

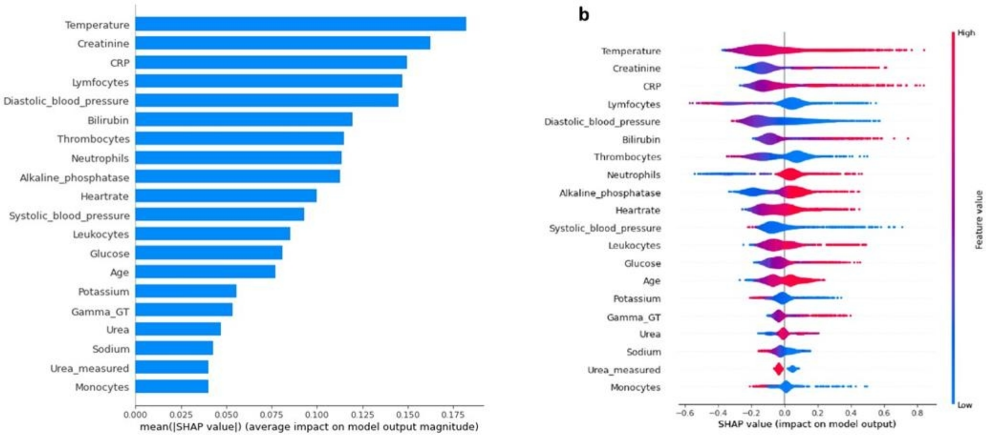

Figure 1 - SHAP values

This figure depicts SHAP (Shapley Additive Explanation) values, illustrating the individual contributions of each variable to the model's prediction. In the left panel (blue bars), we see the average impact of the 20 most important features on the prediction. Meanwhile, the right panel not only shows the feature's contribution but also its direction. Contributions to the left of 0 on the x-axis correspond to negative predictions for blood cultures, while those to the right correspond with positive predictions. Additionally, the color indicates the predictor's actual value, with blue indicating low values and red indicating high values.

277x121mm (96 x 96 DPI)

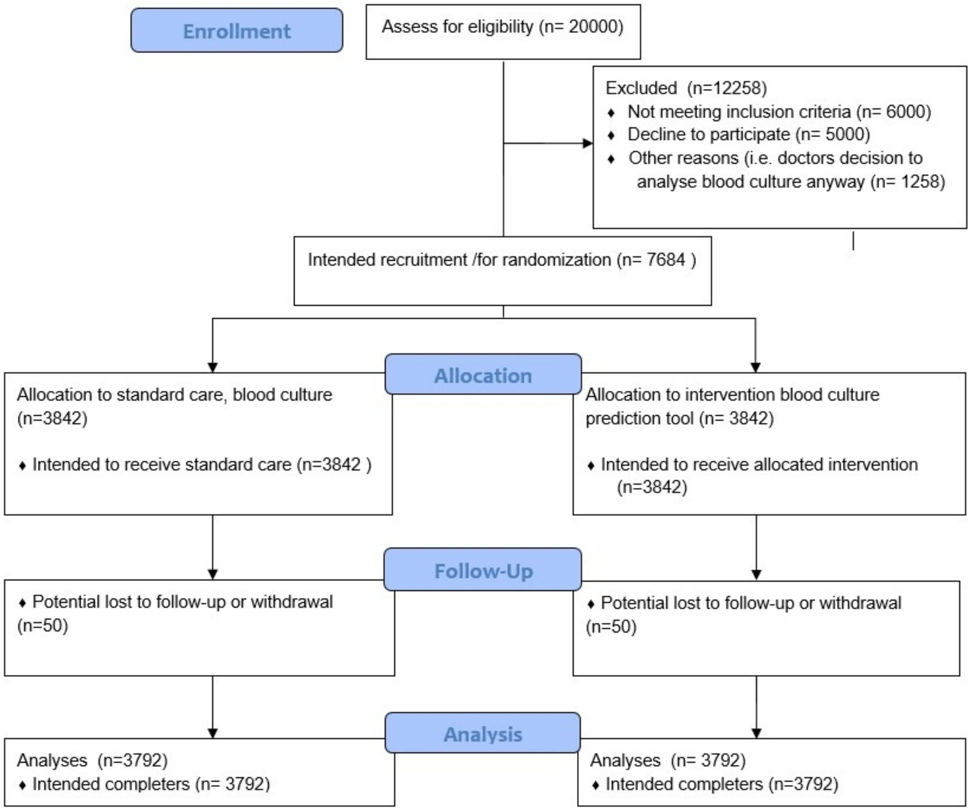

Figure 2 - CONSORT flow chart

223x184mm (96 x 96 DPI)

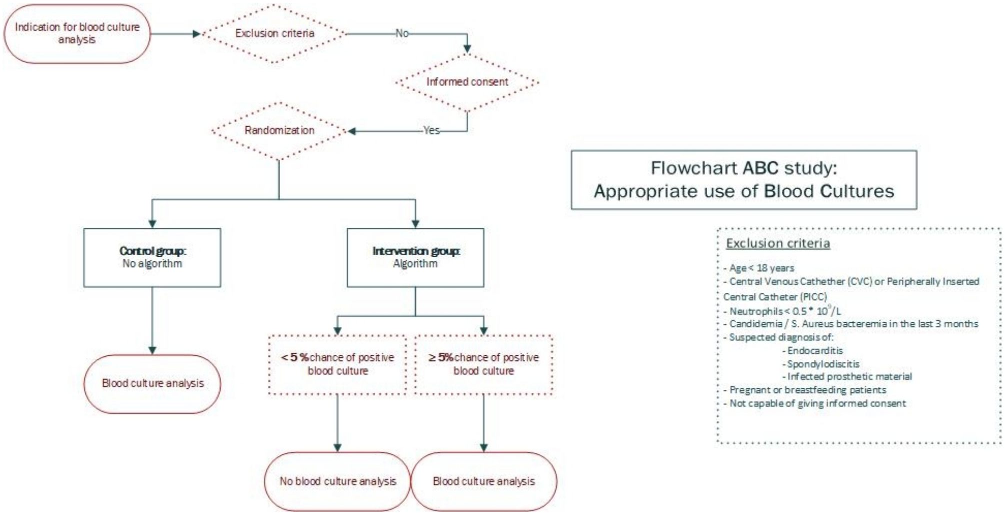

Figure 3 - Flow chart study design

255x130mm (96 x 96 DPI)

Participant Information for Participation in Medical Scientific Research

The ABC Study – Appropriate Use of Blood Cultures in the Emergency Department through Machine Learning: a Randomized Controlled Trial.

Introduction

Dear Sir/Madam,

You are receiving this letter because you have visited the emergency department of Amsterdam UMC and your attending physician intends to collect a blood culture from you, or has already done so. With this information letter, we would like to ask you to participate in medical scientific research. Participation is voluntary. You will learn about the nature of the research, its implications for you, and the advantages and disadvantages involved. It is a lot of information. Would you please read through the information and decide if you wish to participate? If you decide to participate, you can fill out the form provided in the attachment.

Ask Your Questions

You can make your decision based on the information provided in this information letter. Additionally, we recommend the following:

- Ask questions to the researcher who provided you with this information letter.
- Discuss this research with your partner, family, or friends.
- Ask questions to an independent expert
- Read information on www.rijksoverheid.nl/mensenonderzoek.

1. General Information

Amsterdam UMC – location VUmc has initiated this research. Researchers, including physicians, medical students, researchers, and research nurses, conduct the research. The Central Committee on Research Involving Human Subjects (CCMO) has validated this research, and the Medical Ethics Review Committee (METc) has approved it as well. Participation in our scientific research is voluntary. However, we require your written consent to participate.

2. What is the Purpose of the Research?

The aim of this research is to reduce unnecessary diagnostics with blood cultures, since blood cultures can have various adverse effects. Therefore, in this study, we are investigating the safety and effectiveness of a computer program that predicts the outcome of a blood culture. This prediction model assists the physician in deciding whether a blood culture should be analyzed or not. If the probability of the blood culture yielding useful results is very low, we aim to cancel the blood culture analysis and prevent potential adverse effects for the patient.

For peer review only - http://bmjopen.bmj.com/site/about/guidelines.xhtml

3. What is the Background of the Research?

Blood cultures can detect bacteria in the bloodstream. Although bloodstream infections are not common, blood cultures are frequently used as part of standard care for patients with possible infections. In the emergency department, only about 10% of blood cultures yield positive results. The other 90% provide no information or even incorrect information (a bacterium is found, but it is not the cause of the infection). Patients with such "false-positive" cultures often receive unnecessary antibiotics, undergo more procedures, and on average stay in the hospital for a longer period of time.

Our research group has developed a computer program (prediction model) that can predict the likelihood of a positive blood culture. In this study, we aim to omit blood culture analysis when the chance of a positive result is very low, thereby avoiding unnecessary testing. By analyzing fewer blood cultures, we can also reduce the number of false-positive results. Consequently, unnecessary antibiotic administration, procedures, and hospital admissions can be avoided. In this study, we aim to determine whether this approach is indeed safe and assess the benefits for patients.

4. How Does the Research Proceed?

How long does the research last for?

If you participate in the research, your data will be reviewed for up to 3 months after your visit to the emergency department, using the electronic patient record.

Step 1: Are You Eligible to Participate?

We first need to determine if you are eligible to participate. Therefore, the researcher checks whether your attending physician indeed intends to collect a blood culture (or has already done so). The researcher also verifies if there are sufficient data available in your patient record for the prediction model to make a prediction.

In some cases, even if the above conditions apply to you, you may still not be eligible to participate in the research. This is the case, for example, if you recently have had a bloodstream infection.

Step 2: Using the Prediction Model

For this research, we have two groups, and the allocation to these groups is determined by randomization:

• Group 1 (intervention group): The physician of the individuals in this group uses the prediction model.

• Group 2 (control group): The physician of the individuals in this group does not use the prediction model.

Only in Group 1 can the prediction model be used to determine whether the blood culture should be analyzed or not.

For peer review only - http://bmjopen.bmj.com/site/about/guidelines.xhtml

How is it different from standard care?

If you are in the control group (Group 2) of the study, there is no difference from standard care. If you are assigned to Group 1 (the intervention group), your physician may cancel the analysis of your blood culture. Normally, the physician would proceed with the analysis. However, please note that your physician will only make this decision if the prediction model indicates a very low risk for a bloodstream infection. From that point onwards, the physician will consider the blood culture as "negative" and treat you accordingly.

5. What Agreements Do We Make with You?

We want the research to proceed smoothly. Therefore, we make the following agreement with you:

- You will contact the researcher if you no longer wish to participate in the research.

6. What Side Effects, Adverse Effects, or Inconveniences Can You Experience?

The use of the prediction model may lead the physician to not collect a blood culture from you, whereas it would have been done otherwise. Then, there is a very small chance that a bloodstream infection may be missed. Even if such an infection is missed, it is likely to be treated effectively, or you may recover spontaneously. However, in the worst-case scenario, there is a chance of severe harm or even death due to this infection, which could have been prevented by timely treatment based on the result of a blood culture.

7. What are the Benefits and Disadvantages of Participating in the Research?

If you are allocated to the intervention group, your blood culture analysis may be canceled. This reduces unnecessary testing for you. Not only does this save costs, but it may also result in receiving fewer or different antibiotics, undergoing fewer tests, and spending less time in the hospital.

Participating in the research may also have adverse consequences, as mentioned above in point 6. Research on our prediction model has shown that the risk of these adverse consequences is very small.

Do You Not Want to Participate?

You decide whether or not to participate in the research. If you choose not to participate, you will still receive the standard care you would otherwise receive.

8. When Does the Research End?

The researcher will inform you if there is new information about the research that is important for you. The researcher will then ask you if you wish to continue participating. In the following situations, the research ends for you:

- All examinations according to the schedule are completed.
- You decide to withdraw from the research yourself. You can do this at any time. Please inform the researcher immediately. You do not need to explain why you are withdrawing.
- One of the following authorities decides that the research must stop
    - Amsterdam UMC – location VUmc,
    - The government, or the medical ethics committee that assesses the research.

For peer review only - http://bmjopen.bmj.com/site/about/guidelines.xhtml

For peer review only

What Happens If You Withdraw from the Research?

Inform the researcher about this. The researchers will use the data collected up to the point of withdrawal but will not collect any new data.

The entire research is concluded when the data of all participants have been checked in the medical record after 3 months.

9. What Happens After the Research?

Approximately one year after the research is completed, the researchers will publish the results of the research in a medical scientific journal.

10. What Do We Do with Your Data?

If you participate in the research, you also consent to the collection, use, and storage of your data.

What data do we store?

We store this data:

- Your gender

- Your age

- Data about your health

At the end of the research, we will also request your data once from the Personal Records Database (BRP), so that we are informed if anything happens to you outside the hospital.

Why do we collect, use, and store your data?

We collect, use, and store your data to answer the questions of this research and to publish the results.

How do we protect your privacy?

To protect your privacy, we assign a code to your data. Only this code is used on all your data. We keep the key to the code in a secure location at Amsterdam UMC. When processing your data, we always use only this code. Even in reports and publications about the research, no one can tell that it was about you.

Who can see your data?

Some people may view your name and other personal data without a code. These are people who verify whether the researchers are conducting the research properly and reliably. These people can access your data:

• An auditor working for the researcher.

• National and international supervisory authorities. For example, the Healthcare and Youth Inspectorate.

These individuals keep your data confidential. We ask you to give consent for this access.

For peer review only - http://bmjopen.bmj.com/site/about/guidelines.xhtml

How long do we store your data?

We store your data for 15 years in the hospital, to analyze and publish the results.

Can you withdraw your consent for the use of your data?

You can withdraw your consent for the use of your data at any time. This applies to the use in this research and in other research. However, please note: if you withdraw your consent, and researchers have already collected data for a study, they may still use this data.

Can the researchers approach you again for follow-up research?

The researchers may approach you for follow-up research. You can indicate on the consent form whether you agree to this or not.

Do You Want to Know More About Your Privacy?

- Do you want to know more about your rights regarding the processing of personal data? Then visit www.autoriteitpersoonsgegevens.nl.
- Do you have questions about your rights? Or do you have a complaint about the processing of your personal data? Then contact the person responsible for processing your personal data. For your research, that is:
  - Amsterdam UMC
- If you have complaints about the processing of your personal data, we recommend that you first discuss this with the research team. You can also contact the Data Protection Officer of Amsterdam UMC. Or you can file a complaint with the Personal Data Authority.

11. Will You Receive Compensation for Participating in the Research?

You will not receive compensation for participating in this research.

12. Are You Insured During the Research?

Insurance has been taken out for everyone participating in this research. The insurance covers damage caused by the research. But not all damage. In Appendix B, you will find more information about the insurance and the exceptions. It also states to whom you can report damage.

13. Do You Have Questions?

You can ask questions about the research to the research team. If you want advice from someone with no vested interest, consult the independent expert. They are knowledgeable about the research but are not involved in conducting it.

Do you have a complaint? Discuss this with the researcher or the physician treating you. Prefer not to do this? Then contact the hospital's complaints officer. Appendix A provides information on where to find them.

For peer review only - http://bmjopen.bmj.com/site/about/guidelines.xhtml

14. How Do You Give Consent for the Research?

You can take time to consider this research. After an hour of consideration, you inform the researcher whether you understand the information and whether you wish to participate or not. If you choose to participate, you will fill out the consent form provided with this information letter. Both you and the researcher will receive a signed version of this consent statement.

Thank you for your time.

For peer review only

For peer review only - http://bmjopen.bmj.com/site/about/guidelines.xhtml

The ABC Study – 'Appropriate use of Blood Cultures in the emergency department through machine learning: a randomized controlled trial.'

I have read the participant information associated with the ABC study. I understand the information provided. My questions have been answered satisfactorily, and I had sufficient time to decide whether or not to participate in this study. I understand that my participation is entirely voluntary. I am aware that I can withdraw at any time without giving a reason.

• I consent to the use, storage, and processing of my encoded data for research purposes related to blood cultures.

• I consent to the retention of my encoded data for 15 years.

• I understand that for the monitoring of the research, some individuals may access all of my data. These individuals are listed in this information letter. I authorize these individuals to access my data for monitoring purposes.

Please indicate below yes or no:

I consent to potentially being asked to participate in a follow-up study after this research.

Yes ☐ No ☐

I consent to the retrieval of my data from the Personal Records Database (BRP).
Yes ☐ No ☐

Name: ________________________ Signature: __________________

Date: ________________________

I hereby declare that I have fully informed this participant about the ABC study. If any information arises during the study that may affect the participant's consent, I will inform them promptly.

Researcher's Name (or representative): ________________________

Signature: __________________

Date: ________________________

For peer review only - http://bmjopen.bmj.com/site/about/guidelines.xhtml

STANDARD PROTOCOL ITEMS: RECOMMENDATIONS FOR INTERVENTIONAL TRIALS

SPIRIT 2013 Checklist: Recommended items to address in a clinical trial protocol and related documents*

Section/item	Item No	Description	Addressed on page number
**Administrative information**			
Title	1	Descriptive title identifying the study design, population, interventions, and, if applicable, trial acronym	_____1________
Trial registration	2a	Trial identifier and registry name. If not yet registered, name of intended registry	_____2________
	2b	All items from the World Health Organization Trial Registration Data Set	Throughout manuscript
Protocol version	3	Date and version identifier	In original protocol file
Funding	4	Sources and types of financial, material, and other support	______9______
Roles and responsibilities	5a	Names, affiliations, and roles of protocol contributors	______9______
	5b	Name and contact information for the trial sponsor	_____1_______
	5c	Role of study sponsor and funders, if any, in study design; collection, management, analysis, and interpretation of data; writing of the report; and the decision to submit the report for publication, including whether they will have ultimate authority over any of these activities	_______9_____

For peer review only

For peer review only - http://bmjopen.bmj.com/site/about/guidelines.xhtml

	5d	Composition, roles, and responsibilities of the coordinating centre, steering committee, endpoint adjudication committee, data management team, and other individuals or groups overseeing the trial, if applicable (see Item 21a for data monitoring committee)	In original protocol
**Introduction**			
Background and rationale	6a	Description of research question and justification for undertaking the trial, including summary of relevant studies (published and unpublished) examining benefits and harms for each intervention	_____3,4,5____
	6b	Explanation for choice of comparators	______5______
Objectives	7	Specific objectives or hypotheses	______3,5______
Trial design	8	Description of trial design including type of trial (eg, parallel group, crossover, factorial, single group), allocation ratio, and framework (eg, superiority, equivalence, noninferiority, exploratory)	______3,4______
**Methods: Participants, interventions, and outcomes**			
Study setting	9	Description of study settings (eg, community clinic, academic hospital) and list of countries where data will be collected. Reference to where list of study sites can be obtained	______4_______
Eligibility criteria	10	Inclusion and exclusion criteria for participants. If applicable, eligibility criteria for study centres and individuals who will perform the interventions (eg, surgeons, psychotherapists)	______4______
Interventions	11a	Interventions for each group with sufficient detail to allow replication, including how and when they will be administered	______4_____
	11b	Criteria for discontinuing or modifying allocated interventions for a given trial participant (eg, drug dose change in response to harms, participant request, or improving/worsening disease)	Not applicable
	11c	Strategies to improve adherence to intervention protocols, and any procedures for monitoring adherence (eg, drug tablet return, laboratory tests)	Not applicable
	11d	Relevant concomitant care and interventions that are permitted or prohibited during the trial	Not applicable

For peer review only - http://bmjopen.bmj.com/site/about/guidelines.xhtml

Outcomes	12	Primary, secondary, and other outcomes, including the specific measurement variable (eg, systolic blood pressure), analysis metric (eg, change from baseline, final value, time to event), method of aggregation (eg, median, proportion), and time point for each outcome. Explanation of the clinical relevance of chosen efficacy and harm outcomes is strongly recommended	________5_____
Participant timeline	13	Time schedule of enrolment, interventions (including any run-ins and washouts), assessments, and visits for participants. A schematic diagram is highly recommended (see Figure)	______13_____
Sample size	14	Estimated number of participants needed to achieve study objectives and how it was determined, including clinical and statistical assumptions supporting any sample size calculations	________5_____
Recruitment	15	Strategies for achieving adequate participant enrolment to reach target sample size	________5_____

Methods: Assignment of interventions (for controlled trials)

Allocation:

Sequence generation	16a	Method of generating the allocation sequence (eg, computer-generated random numbers), and list of any factors for stratification. To reduce predictability of a random sequence, details of any planned restriction (eg, blocking) should be provided in a separate document that is unavailable to those who enrol participants or assign interventions	_____6______
Allocation concealment mechanism	16b	Mechanism of implementing the allocation sequence (eg, central telephone; sequentially numbered, opaque, sealed envelopes), describing any steps to conceal the sequence until interventions are assigned	_____6_______
Implementation	16c	Who will generate the allocation sequence, who will enrol participants, and who will assign participants to interventions	_____6_______
Blinding (masking)	17a	Who will be blinded after assignment to interventions (eg, trial participants, care providers, outcome assessors, data analysts), and how	_____6_______
	17b	If blinded, circumstances under which unblinding is permissible, and procedure for revealing a participant's allocated intervention during the trial	Not applicable

Methods: Data collection, management, and analysis

Data collection methods	18a	Plans for assessment and collection of outcome, baseline, and other trial data, including any related processes to promote data quality (eg, duplicate measurements, training of assessors) and a description of study instruments (eg, questionnaires, laboratory tests) along with their reliability and validity, if known. Reference to where data collection forms can be found, if not in the protocol	______5______
	18b	Plans to promote participant retention and complete follow-up, including list of any outcome data to be collected for participants who discontinue or deviate from intervention protocols	Not applicable
Data management	19	Plans for data entry, coding, security, and storage, including any related processes to promote data quality (eg, double data entry; range checks for data values). Reference to where details of data management procedures can be found, if not in the protocol	______5______
Statistical methods	20a	Statistical methods for analysing primary and secondary outcomes. Reference to where other details of the statistical analysis plan can be found, if not in the protocol	______5,6_____
	20b	Methods for any additional analyses (eg, subgroup and adjusted analyses)	______6______
	20c	Definition of analysis population relating to protocol non-adherence (eg, as randomised analysis), and any statistical methods to handle missing data (eg, multiple imputation)	______6______

Methods: Monitoring

Data monitoring	21a	Composition of data monitoring committee (DMC); summary of its role and reporting structure; statement of whether it is independent from the sponsor and competing interests; and reference to where further details about its charter can be found, if not in the protocol. Alternatively, an explanation of why a DMC is not needed	______6______
	21b	Description of any interim analyses and stopping guidelines, including who will have access to these interim results and make the final decision to terminate the trial	______6______
Harms	22	Plans for collecting, assessing, reporting, and managing solicited and spontaneously reported adverse events and other unintended effects of trial interventions or trial conduct	_____5,6______
Auditing	23	Frequency and procedures for auditing trial conduct, if any, and whether the process will be independent from investigators and the sponsor	______6______

Ethics and dissemination

For peer review only - http://bmjopen.bmj.com/site/about/guidelines.xhtml

Research ethics approval	24	Plans for seeking research ethics committee/institutional review board (REC/IRB) approval	______7______
Protocol amendments	25	Plans for communicating important protocol modifications (eg, changes to eligibility criteria, outcomes, analyses) to relevant parties (eg, investigators, REC/IRBs, trial participants, trial registries, journals, regulators)	______7______
Consent or assent	26a	Who will obtain informed consent or assent from potential trial participants or authorised surrogates, and how (see Item 32)	______7______
	26b	Additional consent provisions for collection and use of participant data and biological specimens in ancillary studies, if applicable	Not applicable
Confidentiality	27	How personal information about potential and enrolled participants will be collected, shared, and maintained in order to protect confidentiality before, during, and after the trial	______5______
Declaration of interests	28	Financial and other competing interests for principal investigators for the overall trial and each study site	______9______
Access to data	29	Statement of who will have access to the final trial dataset, and disclosure of contractual agreements that limit such access for investigators	______5______
Ancillary and post-trial care	30	Provisions, if any, for ancillary and post-trial care, and for compensation to those who suffer harm from trial participation	Supplement 1
Dissemination policy	31a	Plans for investigators and sponsor to communicate trial results to participants, healthcare professionals, the public, and other relevant groups (eg, via publication, reporting in results databases, or other data sharing arrangements), including any publication restrictions	_____7_______
	31b	Authorship eligibility guidelines and any intended use of professional writers	Not applicable
	31c	Plans, if any, for granting public access to the full protocol, participant-level dataset, and statistical code	______5______
**Appendices**			
Informed consent materials	32	Model consent form and other related documentation given to participants and authorised surrogates	Supplement 1

For peer review only - http://bmjopen.bmj.com/site/about/guidelines.xhtml

Biological specimens	33	Plans for collection, laboratory evaluation, and storage of biological specimens for genetic or molecular analysis in the current trial and for future use in ancillary studies, if applicable	Not applicable

*It is strongly recommended that this checklist be read in conjunction with the SPIRIT 2013 Explanation & Elaboration for important clarification on the items. Amendments to the protocol should be tracked and dated. The SPIRIT checklist is copyrighted by the SPIRIT Group under the Creative Commons "Attribution-NonCommercial-NoDerivs 3.0 Unported" license.

For peer review only
